# CD4^+^ Tregs Drive Post‐Ischemic Sprouting Angiogenesis via Endothelial YY1/MAML1 Reactivation

**DOI:** 10.1002/advs.202518564

**Published:** 2026-05-20

**Authors:** Hang Qu, Cheng Kiu Ho, Jitao Liu, Lei Cui, Yangfeng Hou, Cai Liang, Wenchu Ye, Randolph H. L. Wong, Shohei Hori, Bin Zhou, Kathy O. Lui

**Affiliations:** ^1^ CAS CEMCS‐CUHK Joint Laboratory for Cardiovascular Sciences Department of Chemical Pathology and Li Ka Shing Institute of Health Science The Chinese University of Hong Kong Hong Kong China; ^2^ Department of Surgery Faulty of Medicine Prince of Wales Hospital The Chinese University of Hong Kong Hong Kong China; ^3^ Laboratory of Immunology and Microbiology Graduate School of Pharmaceutical Sciences The University of Tokyo Tokyo Japan; ^4^ CAS CEMCS‐CUHK Joint Laboratory for Cardiovascular Sciences New Cornerstone Science Laboratory Key Laboratory of Multi‐Cell Systems Shanghai Institute of Biochemistry and Cell Biology Center for Excellence in Molecular Cell Science Chinese Academy of Sciences Shanghai China

**Keywords:** angiogenesis, CD4^+^ Tregs, endothelial cells, MAML1, YY1

## Abstract

Microvascular complications of diabetes are chronic diseases of small vessels. We previously found that CD4^+^ regulatory T‐cells (Tregs) are markedly reduced in type 2 diabetes (T2D) after ischemic injury in both mice and humans, and that Treg deficiency in immunodeficient mice impairs vascular regeneration. However, the mechanisms by which Tregs protect against diabetic vascular disease remain unclear. Here, we demonstrate that endothelial cells (ECs) upregulate the transcription factor YY1 during post‐ischemic vascular regeneration, but this response is blunted in diabetic ECs. Endothelial‐specific deletion of YY1 leads to defective vascular regeneration following ischemia. Mechanistically, YY1 binds to the *MAML1* promoter at regions enriched for H3K4me3 and H3K27ac. YY1 activates *MAML1* transcription, likely by recruiting H3K4me3 writers (SETD1A, MLL1, MLL2) and their scaffold WDR5 within the COMPASS complex, together with the H3K27ac writer p300. Functionally, Tregs enhance vascular regeneration through paracrine signaling even in T2D mice. Adoptive Treg transfer restores regenerative capacity by reactivating the endothelial YY1/MAML1 axis. These findings identify a Treg‐YY1‐MAML1 pathway as a key regulator of endothelial function and vascular repair, offering mechanistic insight into how Tregs promote tissue regeneration in diabetes‐associated microvascular dysfunction.

## Abstract 2

In healthy condition, ischemia recruits CD4^+^ Tregs that paracrinally induce endothelial YY1. YY1 recruits SETD1A and p300 to epigenetically activate the *Maml1* promoter via H3K4me3 and H3K27ac, driving regeneration. In diabetes, ischemia fails to recruit CD4^+^ Tregs, blunting this pathway. Thus, a CD4^+^ Treg–YY1–MAML1 axis governs endothelial programs and angiogenesis, suggesting new therapies for PAD in obesity/diabetes.

## 1 Introduction

Angiogenesis, the formation of new blood vessels, is critical for development and tissue repair. We previously demonstrated that VEGF‐modified mRNA can induce angiogenesis and promote regeneration in the heart [1] and pancreas [2]. By contrast, angiogenesis in the lower extremities is markedly impaired in peripheral arterial disease (PAD). At its most severe stage, critical limb‐threatening ischemia carries a >50% 5‐year mortality rate and a high risk of amputation [3]. In healthy individuals, ischemia triggers sprouting angiogenesis, but this response is blunted in PAD, particularly in patients with obesity and diabetes. Our prior work showed that CD4^+^ Th1 cells and CD8^+^ T cells suppress vascular growth after hindlimb ischemia under hyperglycemic conditions; blocking their activation with therapeutic monoclonal antibodies enhances vascular regeneration [4, 5]. Notably, CD4 blockade expands CD4^+^ regulatory T cells (Tregs), which promote sprouting angiogenesis, characterized by increased apelin expression, even in type‐2 diabetes (T2D) [4]. Despite these advances, the precise mechanisms by which CD4^+^ Tregs exert pro‐angiogenic programs in diabetic PAD remain unclear. Elucidating these pathways is critical for developing clinically relevant strategies to restore vascular repair in diabetes‐associated vascular dysfunction.

Yin Yang 1 (YY1) is a ubiquitously expressed GLI‐Kruppel‐family zinc finger transcription factor with indispensable roles in gene regulation across diverse biological systems. Functioning primarily as a sequence‐specific DNA‐binding protein, YY1 can activate or repress target genes, with its regulatory effects shaded by cellular context, including cell type, signaling pathways, and interactions with co‐regulators. In pancreatic beta cells, we showed that *Yy1* depletion causes type‐1 diabetes, reflecting YY1's role in binding enhancer regions of *Ins* genes and facilitating insulin production by stabilizing enhancer‐promoter interactions through RNA polymerase II [6]. In vascular smooth muscle cells (VSMCs), we demonstrated that *Yy1* ablation causes hypotension due to its regulation of *Mettl3* transcription, thereby altering m^6^A‐dependent transcript stability of contractile genes such as *Myh11*, *Mylk2* and *Tgfb2* and impairing vascular tone and blood pressure responses to dynamic stimuli [7]. In ventricular myocytes, YY1 activates *Mettl1*, promoting *Srsf9* expression through m^7^G modification during hypertrophic remodeling [8]. In endothelial cells (ECs), YY1 was first nominated by ISMARA, a web‐based platform for genome‐wide promoter activity analysis, without functional validation, as the top‐ranked transcriptional regulator of sprouting angiogenesis during retinal development [9]. Subsequent studies provided functional evidence that endothelial *Yy1* depletion results in embryonic lethality with defective sprouting angiogenesis and impaired vascular maturation [10]. Mechanistically, YY1 represses Notch signaling by directly interacting with the transcription factor RBPJ to form a nuclear YY1‐RBPJ repressor complex, thereby preventing RBPJ from binding with the Notch coactivator MAML1. Although the precise mechanisms by which the YY1‐RBPJ repressor complex governs sprouting angiogenesis remain to be fully defined, current data underscore a critical role for YY1 in vascular development (for review, see [11]). Collectively, these studies highlight YY1's capability to regulate gene expression directly at the DNA level, indirectly through epitranscriptomic mechanisms, and via competitive interactions with other transcription factors. Nevertheless, the upstream cellular drivers of YY1 activity remain unknown.

In this study, we identified endothelial YY1 as a mediator through which CD4^+^ Tregs drive angiogenesis in non‐diabetic condition. Following ischemic injury, endothelial YY1 is upregulated in normoglycemic mice and humans but is attenuated in diabetes. Unlike its role in early development, YY1 in adult ECs does not interact with RBPJ; instead, it promotes MAML1 expression by recruiting SETD1A and p300 to the *Maml1* promoter to facilitate H3K4 trimethylation and H3K27 acetylation. Functionally, overexpression of MAML1 via AAV improved vascular reperfusion after ischemia in YY1 deficient mice. We previously reported that ischemia induces CD4^+^ Tregs in limbs undergoing vascular regeneration but not in diabetic mice [4]. Here, we show that ischemia also increases YY1 expression and occupancy at *Maml1* promoter in regenerating vessels. To test causality, we performed adoptive transfer of CD4^+^ Tregs, which enhanced vascular regeneration even in diabetic mice; this effect was markedly reduced by endothelial *Yy1* ablation. Conversely, depletion of CD4^+^ Tregs in *NOD.Foxp3^hCD2^* mice through lytic anti‐hCD2 antibodies reduced YY1 expression and impaired vascular regeneration. CD4^+^ Tregs or their conditioned supernatant upregulate YY1 and MAML1 in ECs, and the paracrine factors GAS6, GRN, and AREG each increase YY1/MAML1 expression through their respective receptor activation. These findings reveal a CD4^+^ Treg‐YY1‐MAML1 axis that regulates endothelial behavior and in vivo transcriptional control of angiogenesis after ischemic injury, offering new therapeutic opportunities for PAD in obesity and diabetes.

## 2 Results

### 2.1 YY1 is Markedly Upregulated in Healthy ECs, but not in Diabetic ECs, After Ischemic Injury

**FIGURE 1:**
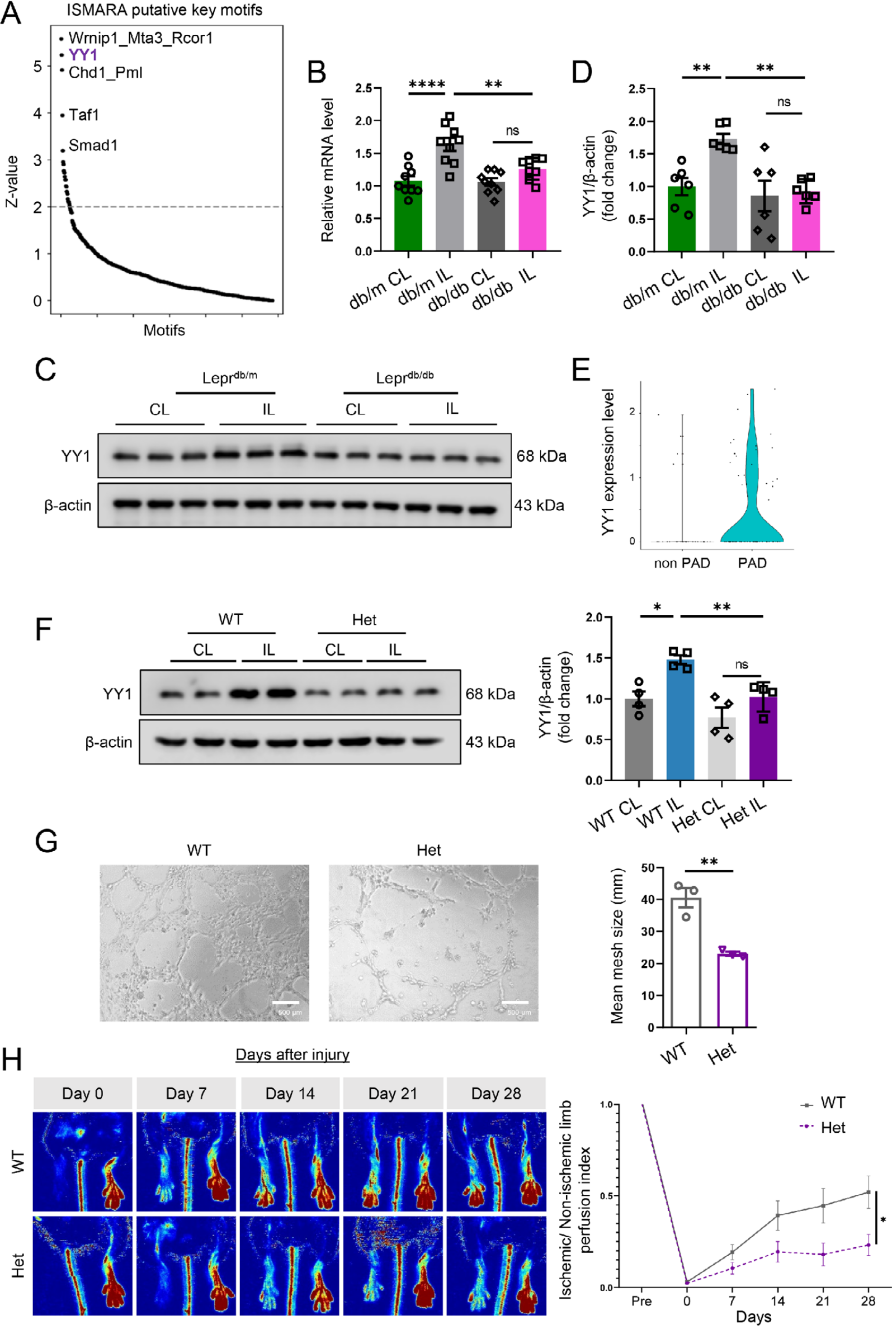
Endothelial YY1 is essential in vascular regeneration after ischemic injury (A) Genome‐wide promoter activity analyzed by Integrated System for Motif Activity Response Analysis (ISMARA) demonstrating the transcription factors that most actively bind to common motifs shared by target genes as identified by RNA‐sequencing. (B) Quantitative real‐time PCR analysis of *Yy1* mRNA expression in the ischemic limb (IL) and control limb (CL) of and *Lepr^db/+^* and *Lepr^db/db^* mice. Statistical analysis was performed using two‐way ANOVA with *Tukey's* multiple comparisons test (n = 8–9). **p < 0.01, ****p < 0.0001, (ns, no significant difference). (C) Western blot detection of YY1 protein expression in IL and CL of *Lepr^db/+^* and *Lepr^db/db^* mice, with β‐actin as an internal control. (D) Quantitative ratio of YY1/β‐actin in (C). *p < 0.05 (ns, no significant difference) (n = 3). Data were analyzed via two‐way ANOVA with *Tukey's* multiple comparisons test. (E) Violin plots of YY1 expression in CD45^−^CD31^+^CD144^+^ ECs from gastrocnemius muscle of non‐diabetic patients with lower‐extremity PAD versus non‐PAD controls. (F) Western blot analysis of YY1 protein expression in IL and CL of *Yy1^fl/fl^* (WT) and *Cdh5‐Cre;Yy1^fl/+^* (Het) mice, with β‐actin as an internal control. The right panel shows the quantitative ratio of YY1/β‐actin. *p < 0.05, **p < 0.01 (n = 3–5). Data were analyzed via two‐way ANOVA with *Tukey's* multiple comparisons test. (G) Microscopic images of primary endothelial cells isolated from *Yy1^fl/fl^* (WT) and *Cdh5‐Cre;Yy1^fl/+^* (Het) mice. The right panel shows the quantitative of mean mesh size. *p < 0.05, **p < 0.01 (n = 3). (H) Representative Laser Doppler images and quantification of hindlimb blood perfusion *Yy1^fl/fl^* (WT) and *Cdh5‐Cre;Yy1^fl/+^* (Het). Pre: pre‐ischemia baseline. Statistical analysis was performed using two‐way ANOVA with Sidak's post‐hoc test. *p < 0.05 (n = 5–6). Data are presented as mean ± S.E.M. . Scale bar = 500 µm. Each dot represents 1 biological replicate. The *p* values were calculated by unpaired two‐tailed *t*‐test (G, H) or two‐way ANOVA with *Tukey's* multiple comparisons test (B, D, F).

We previously reported that ECs from Lepr^db/db^ mice recapitulate key features of PAD in T2D patients, exhibiting a pro‐inflammatory phenotype and impaired post‐ischemic vascular regeneration compared with Lepr^db/+^ littermate controls [4]. To investigate the basis of this regenerative deficit, we profiled transcriptional regulators of EC behavior using cells isolated from ischemic tissues following femoral artery ligation. Genome‐wide promoter activity was inferred from bulk RNA‐seq data using ISMARA, as previously described [9]. Based on significantly altered motif activities between groups (z value > 2), *Yy1* emerged among the highest scoring candidates with known endothelial expression (Figure 1A). Additional endothelial regulators, such as Chd1 [12], Pml [13], Taf1 [14] and Smad1 [15], were also identified. Although YY1 has been implicated in normal EC development, its role in diabetic vascular disease has remained unclear. To test this, we performed RT‐qPCR and Western blotting on ECs isolated from ischemic tissues 10 days after ligation of the femoral artery in Lepr^db/db^ and control mice. In non‐diabetic ECs, the mRNA and protein levels of *Yy1*/YY1 were significantly increased after ischemia, whereas levels remained unchanged in diabetic ECs (Figure 1B–D). To assess translational relevance, we analyzed a published single‐nuclei RNA‐seq dataset [16]. Among CD45^−^CD31^+^CD144^+^ ECs in gastrocnemius muscle, *YY1* was also upregulated in non‐diabetic patients with lower extremity PAD compared with those without PAD (Figure 1E). These findings suggest a failure of endothelial YY1 to respond to ischemic injury in diabetes.

### 2.2 Endothelial YY1 is Essential in Vascular Regeneration After Ischemic Injury

A previous study revealed that endothelial YY1 is essential for vascular development, as *Cdh5‐Cre;Yy1^fl/fl^* embryos are lethal [10]. This early lethality limits functional studies of endothelial YY1 beyond the postnatal stage. To examine YY1 in adult ECs, we generated heterozygous *Cdh5‐Cre;Yy1^fl/+^* mice, which survived normally compared with *Yy1^fl/fl^* controls. Western blotting showed that heterozygous mutants also failed to upregulate endothelial YY1 after ischemia (Figure 1F). To investigate whether YY1 drives angiogenesis, we knocked down *YY1* in human embryonic stem cell‐derived ECs (hESC‐ECs) using siRNA and observed markedly impaired tube formation, reflected by reduced area, length, numbers of nodes and junctions (Figure S1A). Consistently, purified lung ECs from *Cdh5‐Cre;Yy1^fl/+^* mice showed significantly reduced angiogenesis compared with controls (Figure 1G). To determine the impact of endothelial YY1 on functional reperfusion, we performed laser Doppler imaging weekly for 4 weeks after ischemic injury and found a significant reduction in blood flow recovery in the ischemic limbs of *Cdh5‐Cre;Yy1^fl/+^* mice than controls (Figure 1H). To circumvent developmental lethality, we also generated *Cdh5‐CreERT;Yy1^fl/fl^* mice. Given that tamoxifen‐activated *CreERT* can impair angiogenesis independently of gene deletion [17], *Cdh5‐CreERT* mice were used as controls. Two weeks after the last dose of tamoxifen, mice underwent ischemic injury and were monitored weekly by laser Doppler imaging. Similarly, *Cdh5‐CreERT;Yy1^fl/fl^* mice exhibited significantly reduced reperfusion compared with controls (Figure S1B). Collectively, these findings indicate that endothelial YY1 is required for promoting vascular regeneration after ischemic injury.

### 2.3 Endothelial YY1 Inhibits Inflammation and Regulates Notch Signaling in Adult ECs

**FIGURE 2:**
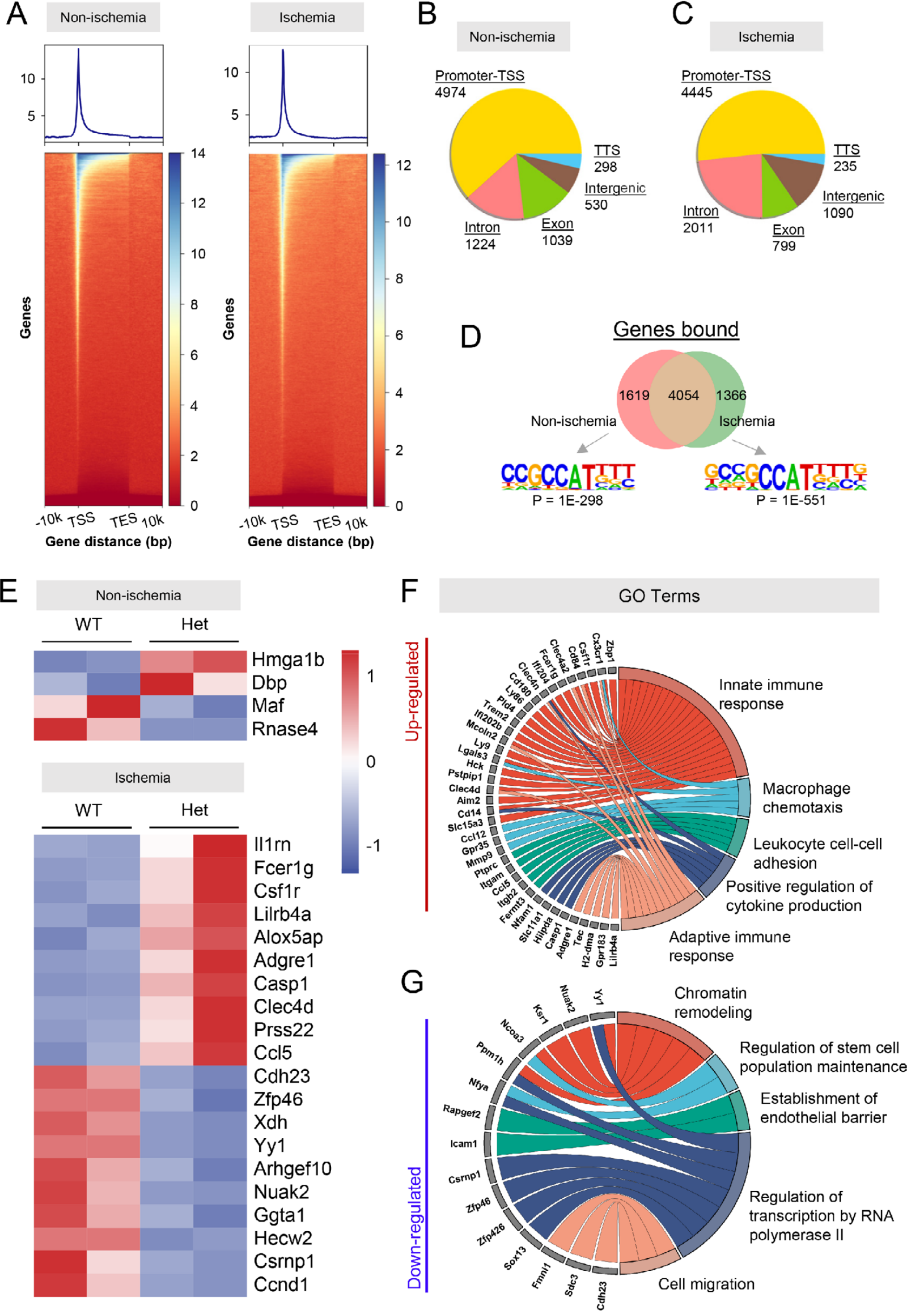
Endothelial YY1 inhibits inflammation in adult ECs (A) Heatmaps representing the normalized YY1 ChIP‐seq intensities ± 10 kb over the gene body from transcription start site (TSS) to transcription end site (TES) of YY1‐bound DNA in ECs from non‐ischemic (left panel) and ischemic (right panel) limbs. The upper panels show the enrichment plots representing the average distribution of YY1 intensities ± 10 kb over the gene body. (B, C) Typical peak annotation pie chart shows that the majority of the peaks fall into promoter/TSS regions in ECs from both non‐ischemic and ischemic limbs. (D) Venn diagram summarizing the numbers of protein‐coding genes bound by YY1 in ECs from non‐ischemic and ischemic limbs. *De novo* motifs were identified by HOMER. (E) (Upper panel) Heatmap of the 2 significantly upregulated and 2 significantly downregulated genes with YY1 bound peaks in ECs from non‐ischemic limbs after Yy1 ablation. (Lower panel) Heatmap of top 10 significantly upregulated and top 10 significantly downregulated genes with YY1 bound peaks in ECs from ischemic limbs after Yy1 ablation. (F, G) Gene Ontology (GO) enrichment analysis of differentially expressed genes in ECs from ischemic limbs after Yy1 ablation.

Next, we examined the molecular mechanisms by which endothelial YY1 promotes vascular regeneration after ischemic injury. To identify direct targets, we performed YY1 ChIP‐seq on CD31^+^ ECs isolated from ischemic and non‐ischemic limbs of *Yy1^fl/fl^* control mice 7 days after femoral artery ligation (Figure 2A). YY1 binding was enriched at promoter‐transcription start sites (TSS) both before (Figure 2B) and after (Figure 2C) ischemia; however, ischemia shifted the binding landscape, decreasing enrichment at promoter‐TSS and exonic regions while increasing occupancy at intergenic and intronic regions (Figure 2B,C). Consistent with reports in pancreatic beta cells [6] and VSMCs [7], *de novo* motif analysis identified GCCAT as the core YY1‐binding motif in adult ECs, presented in 26.61% of peaks before ischemia (*p* = 1 × 10^−298^) and 35.13% after ischemia (*p* = 1 × 10^−551^) (Figure 2D). We identified 1619 protein‐coding genes uniquely targeted in ECs from non‐ischemic limbs and 1366 uniquely targeted in those from ischemic limbs (Figure 2D).

To identify candidates mediating YY1‐dependent vascular function and regeneration, we respectively isolated CD31^+^ ECs from ischemic and non‐ischemic limbs of *Cdh5‐Cre;Yy1^fl/+^* mice and *Yy1^fl/fl^* controls 7 days after femoral artery ligation for bulk RNA‐seq. We then integrated YY1 ChIP‐seq targets with the corresponding RNA‐seq datasets: 1,619 YY1‐bound genes from non‐ischemic limbs with the non‐ischemic RNA‐seq, and 1,366 YY1‐bound genes from ischemic limbs with the ischemic RNA‐seq. In ECs from non‐ischemic limbs, YY1 ablation yielded only 4 differentially expressed genes with YY1 peaks: 2 significantly upregulated (*Hmga1b*, *Dbp*) and 2 significantly downregulated (*Maf*, *Rnase4*) (Figure 2E). In contrast, in ECs from ischemic limbs, YY1 ablation resulted in 119 significantly upregulated and 49 downregulated genes with YY1 peaks. Among the top ten most significantly upregulated genes were myeloid pathway genes (*Il1rn*, *Fcer1g*, *Csf1r*, *Lilrb4a*, *Alox5ap*, *Adgre1*, *Casp1*, *Clec4d*) and inflammatory modulators (*Il1rn*, *Ccl5*), whereas the top ten most significantly downregulated genes involved in cell proliferation (*Zfp46*, *Arhgef10*, *Ccnd1*) (Figure 2E). GO enrichment analysis of the upregulated genes after YY1 depletion highlighted biological pathways related to the innate immune response, macrophage chemotaxis, leukocyte cell‐cell adhesion, positive regulation of cytokine production, and adaptive immune response (Figure 2F and Table S1). Conversely, downregulated genes were enriched for chromatin remodeling, regulation of stem cell population maintenance, establishment of endothelial barrier, regulation of transcription by RNA pol II, and cell migration (Figure 2G and Table S2).

**FIGURE 3:**
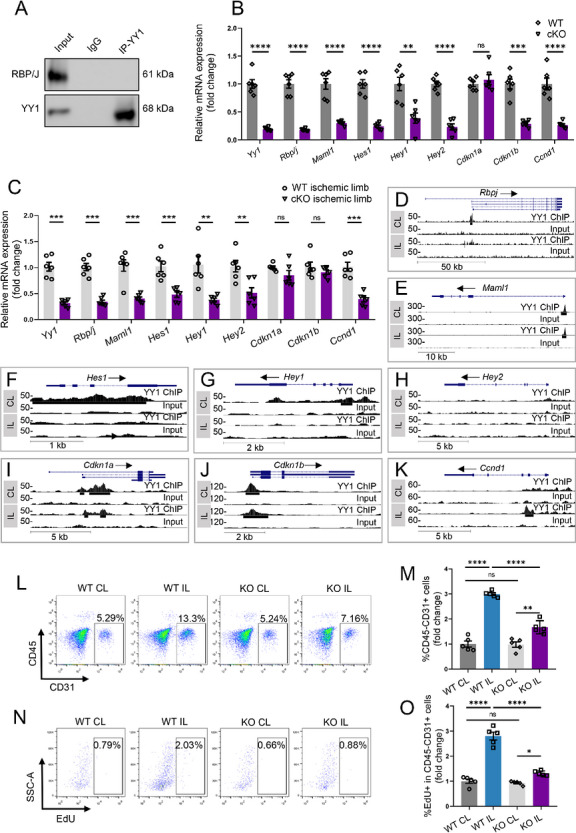
Endothelial YY1 enhances Notch signaling in adult ECs (A) Co‐IP assay validating YY1‐RBP/J interaction. (B) Quantitative real‐time PCR analysis of *Rbpj, Maml1, Hes1, Hey1, Hey2, Cdkn1a, Cdkn1b and Ccnd1* mRNA expression in *Yy1^fl/fl^* (WT) and *Cdh5‐Cre;Yy1^fl/fl^* (cKO) from E11.5 embryo CD31^+^ cells. **p < 0.01, ***p < 0.001, ****p < 0.0001, (ns, no significant difference). (n = 6). (C) Quantitative real‐time PCR analysis of *Rbpj, Maml1, Hes1, Hey1, Hey2, Cdkn1a, Cdkn1b and Ccnd1* mRNA expression in *Cdh5‐CreER* (WT) and *Cdh5‐CreER;Yy1^fl/fl^* (cKO) from ischemic limb CD31+ cells at 14 days after ischemia. **p < 0.01, ***p < 0.001, (ns, no significant difference). (n = 6). (D—K) Genome snapshot from YY1 ChIP‐seq analysis using ECs from non‐ischemic and ischemic limbs at *Rbpj, Maml1, Hes1, Hey1, Hey2, Cdkn1a, Cdkn1b and Ccnd1* gene loci respectively. (L) Representative flow cytometry plots for CD45^−^CD31^+^ cells from the *Cdh5‐CreER* (WT) and *Cdh5‐CreER;Yy1^fl/fl^* (cKO) hindlimb muscle CL and IL respectively. (M) Quantification of CD45^−^CD31^+^ cells in (L). Data are mean ± SEM. Data were analyzed via two‐way ANOVA with *Tukey's* multiple comparisons test. **p < 0.01, ****p < 0.0001, (ns, no significant difference). (n = 5). (N) Representative flow cytometry plots for EdU^+^ cells in CD45^−^CD31^+^ cells gated from (L). (O) Quantification of EdU^+^ cells in (N). Data are mean ± SEM. Data were analyzed via two‐way ANOVA with *Tukey's* multiple comparisons test. *p < 0.05, ****p < 0.0001, (ns, no significant difference). (n = 5). Data are presented as mean ± S.E.M. . Each dot represents 1 biological replicate. The *p* values were calculated by unpaired two‐tailed *t*‐test (B, C) or two‐way ANOVA with *Tukey's* multiple comparisons test (M, N).

A prior study reported that YY1 represses Notch signaling by interacting with RBPJ and prevents its binding to MAML1 during embryonic angiogenesis [10]. To determine whether a similar mechanism operates in adult angiogenesis, we performed co‐immunoprecipitation (co‐IP) in adult ECs to assess YY1‐RBPJ interactions. Unexpectedly, no interaction was detected (Figure 3A). We next examined Notch regulators and targets in embryonic and adult post‐ischemic CD31^+^ ECs. Compared with *Yy1^fl/fl^* controls, CD31^+^ ECs from *Cdh5‐Cre;Yy1^fl/fl^* embryos at embryonic day (E) 11.5 exhibited significant reductions in the regulators *Rbpj* and *Maml1*, as well as in the Notch targets *Hes1*, *Hey1*, *Hey2*, *Cdkn1b*, and *Ccnd1* (Figure 3B). Consistently, most of these transcripts were also significantly reduced in CD31^+^ ECs from *Cdh5‐CreER;Yy1^fl/fl^* adults after ischemia compared to *Cdh5‐CreER* controls (Figure 3C). Notably, *Hey1* was significantly reduced in both embryonic ECs and adult post‐ischemic ECs upon *Yy1* ablation (Figure 3B,C), in contrast to its increase in *Yy1*‐deficeint ECs reported previously [10]. To clarify how YY1 influences Notch signaling without detectable RBPJ interaction in adult ECs, we analyzed YY1 ChIP‐seq. Before ischemia, YY1 showed robust occupancy at multiple Notch‐pathway genes, including *Rbpj*, *Maml1*, *Hes1*, *Hey1*, *Cdkn1a*, and *Cdkn1b* (Figure 3D–G,I,J). Following ischemia, enrichment was retained primarily at *Maml1*, *Cdkn1a*, and *Cdkn1b*, with new binding at *Ccnd1* (Figure 3E,I–K), indicating a redistribution from canonical Notch targets toward cell‐cycle regulators. Functionally, we performed hindlimb ischemia 2 weeks after tamoxifen, injected EdU on day 13, and on day 14 quantified CD45^−^CD31^+^EdU^+^ ECs by flow cytometry. Both the % CD45^−^CD31^+^ cells and endothelial proliferation (EdU^+^) significantly increased in control mice after ischemia (Figure 3L–O), whereas this proliferative response was drastically reduced in YY1‐deficient mice (cKO IL vs WT IL). Altogether, these data support a direct, cell‐autonomous role for endothelial YY1, potentially acting via MAML1 and cell‐cycle effectors, in promoting vascular regeneration after injury, independent of RBPJ in adult ECs.

### 2.4 Endothelial YY1 Directly Regulates *Maml1* Through Histone Modifications

**FIGURE 4:**
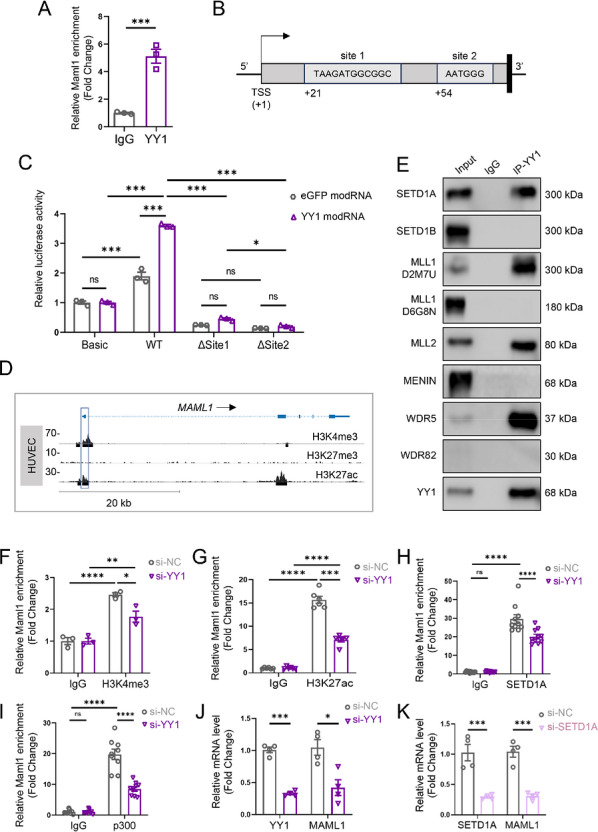
Endothelial YY1 directly regulates *Maml1* through histone modifications (A) ChIP‐quantitative PCR analysis for YY1 binding to the putative *MAML1* promoter. Chromatin was extracted from hESC‐ECs and then precipitated with an anti‐YY1 antibody or IgG (negative control). ***p < 0.001. (n = 3). (B) Schematic diagram illustrating the two predicted binding sites and the sequences deleted in the two mutant reporters, with the locations shown relative to the transcription start site (TSS). (C) Dual‐luciferase reporter assay for the MAML1 promoter. Plasmids include pGL3‐Basic, pGL3‐wtMAML1 promoter (WT), and deletion mutants (Del1/ΔSite1, Del2/ΔSite2). *p < 0.05, ***p < 0.001, (ns, no significant difference). (n = 3). (D) Genome snapshot of H3K4me3, H3K27me3 and H3K27ac ChIP‐seq using human umbilical vein endothelial cells at MAML1 gene locus. (E) Co‐IP showing the endogenous protein interaction between YY1 and H3K4me3 writers or co‐factors in hESC‐ECs. Protein was extracted and then precipitated with an anti‐YY1 antibody or IgG before immunoblot. (F–I) ChIP‐quantitative PCR analysis for H3K4me3, H3K27ac, SETD1A or p300 binding to the putative *MAML1* promoter after being treated with YY1 siRNA. Chromatin was extracted from hESC‐ECs and then precipitated with an anti‐ H3K4me3, H3K27ac, SETD1A or p300 antibody or IgG (negative control). *p < 0.05, **p < 0.01, ***p < 0.001, ****p < 0.0001, (ns, no significant difference). (n = 3–9). (J) Quantitative real‐time PCR analysis of *YY1 MAML1* mRNA expression in hESC‐ECs after being treated with YY1 siRNA. *p < 0.05, ***p < 0.001, (n = 4). (K) Quantitative real‐time PCR analysis of *SETD1A MAML1* mRNA expression in hESC‐ECs after being treated with SETD1A siRNA. ***p < 0.001, (n = 4). Data are presented as mean ± S.E.M. . Each dot represents 1 biological replicate. The *p* values were calculated by unpaired *t*‐tests (A, J, K), two‐way ANOVA with *Tukey's* multiple comparisons test (C, F, G, H, I).

Following ischemia, YY1 showed enriched binding at the Notch regulator/target genes *Maml1*, *Cdkn1a*, *Cdkn1b* and *Ccnd1*. Among these, MAML1 has yet been established as a downstream target of YY1. MAML1 is a rate‐limiting transcriptional coactivator in Notch signaling that bridges the Notch intracellular domain (NICD)‐RBPJ complex to additional coregulators, including p300/CBP, to enable robust transcriptional activation [18, 19]. We, therefore, investigated how YY1 facilitates *MAML1* transcription. Guided by the YY1 ChIP‐seq peak at the *Maml1* promoter (Figure 3E), bioinformatic analysis identified a conserved YY1‐binding motif (CCAT; ATGG on the reverse strand) within the MAML1 promoter across mouse and human genomes (Figure S2). ChIP‐qPCR confirmed increased YY1 occupancy at the *MAML1* promoter in hESC‐ECs (Figure 4A). To examine functional relevance, we conducted firefly luciferase reporter assays. A 506‐bp fragment of the human *MAML1* promoter (hg38 chr5:179,732,707 – 179,733,212) was cloned upstream of luciferase in pGL3‐basic to generate the wildtype (WT) reporter. Two predicted YY1 binding sites were targeted by deletion: a 12‐bp sequence at site 1 (Del1/ ΔSite1) and a 6‐bp sequence at site 2 (Del2/ ΔSite2), with locations shown related to the TSS (Figure 4B and Figure S3). To test whether YY1 regulates *MAML1* promoter activity via these motifs, we compared WT, Del1, and Del2 reporters in hESC‐ECs transfected with YY1 or eGFP modRNA (Figure 4C). YY1 overexpression significantly increased WT promoter activity (YY1 vs. eGFP, WT) but had no effect on either deletion reporter (YY1 vs. eGFP, Del1 or Del2). Consistently, Del1 and Del2 activities did not differ between YY1 and eGFP groups, indicating that removal of either site abolishes YY1 responsiveness. Moreover, in the presence of YY1 modRNA, luciferase activity was significantly lower for Del2 than for Del1 (Del2< Del1; p < 0.05), indicating a larger loss of YY1‐dependent activation when site 2 was removed. These data suggest that site 2 is the predominant contributor to YY1‐mediated regulation of the *MAML1* promoter. A two‐way ANOVA (Construct × Treatment) revealed a significant interaction, consistent with YY1‐dependent transactivation that requires intact motifs. These results support motif‐dependent regulation of the *MAML1* promoter by YY1.

Next, we investigated how YY1 activates *MAML1* transcription by analyzing global histone modifications in human ECs using published datasets: H3K4me3 for primed promoters, H3K27me3 for poised enhancers, and H3K27ac for active enhancers. YY1 bound the *MAML1* promoter adjacent to H3K4me3 and H3K27ac marks (Figure 4D). Considering the proximity of YY1 binding sites to H3K4me3‐ and H3K27ac‐enriched regions, we examined whether YY1 mediates histone modifications at the *MAML1* promoter by recruiting histone methyltransferase and acetyltransferase writers. Co‐IP assays in hESC‐ECs showed that YY1 interacted with SETD1A, MLL1, MLL2, and the cofactor WDR5 (Figure 4E). YY1 also bound the histone acetyltransferase p300 (Figure S4A), an interaction we confirmed in murine lung ECs using YY1 immunoprecipitation (Figure S4B). We hypothesized that YY1 enriches the transcription initiation complex by forming a YY1‐SETD1A/p300‐RNA pol II assembly. Consistently, YY1 directly interacted with pol II in hESC‐ECs (Figure S4A) and murine lung ECs (Figure S4B). In hESC‐ECs, *YY1* knockdown by siRNA (Figure S4C,D) followed by ChIP‐qPCR at 48 h post knockdown revealed significantly reduced H3K4me3 and H3K27ac at the *MAML1* promoter (Figure 4F, G), indicating that YY1 is required for these activating histone marks, possibly through recruiting the SETD1A/p300‐RNA pol II complex. Consistently, *YY1* knockdown diminished SETD1A and p300 occupancy at the *MAML1* promoter (Figure 4H, I), and knockdown of *YY1* or *SETD1A* significantly decreased *MAML1* mRNA (Figure 4J, K). We also observed a direct YY1‐MAML1 interaction (Figure S4E), suggesting that YY1 can act as a MAML1 cofactor in Notch activation. In mice, CUT&RUN‐seq revealed YY1 binding at the *Maml1* promoter adjacent to H3K4me3 and H3K27ac (Figure S5A). Fourteen days after ischemia, ECs isolated by CD31 beads exhibited increased YY1 interactions with SETD1A, p300 and RNA pol II (Figure S5B), and increased YY1 enrichment at the *Maml1* promoter (Figure S5C). Together, these findings support a model in which YY1 functions within a SETD1A/p300‐RNA pol II transcription initiation complex to promote *MAML1* transcription by epigenetically promoting H3K4 trimethylation and H3K27 acetylation at its promoter—an effect amplified by ischemia via increased YY1 expression, promoter occupancy, and cofactor interactions.

**FIGURE 5:**
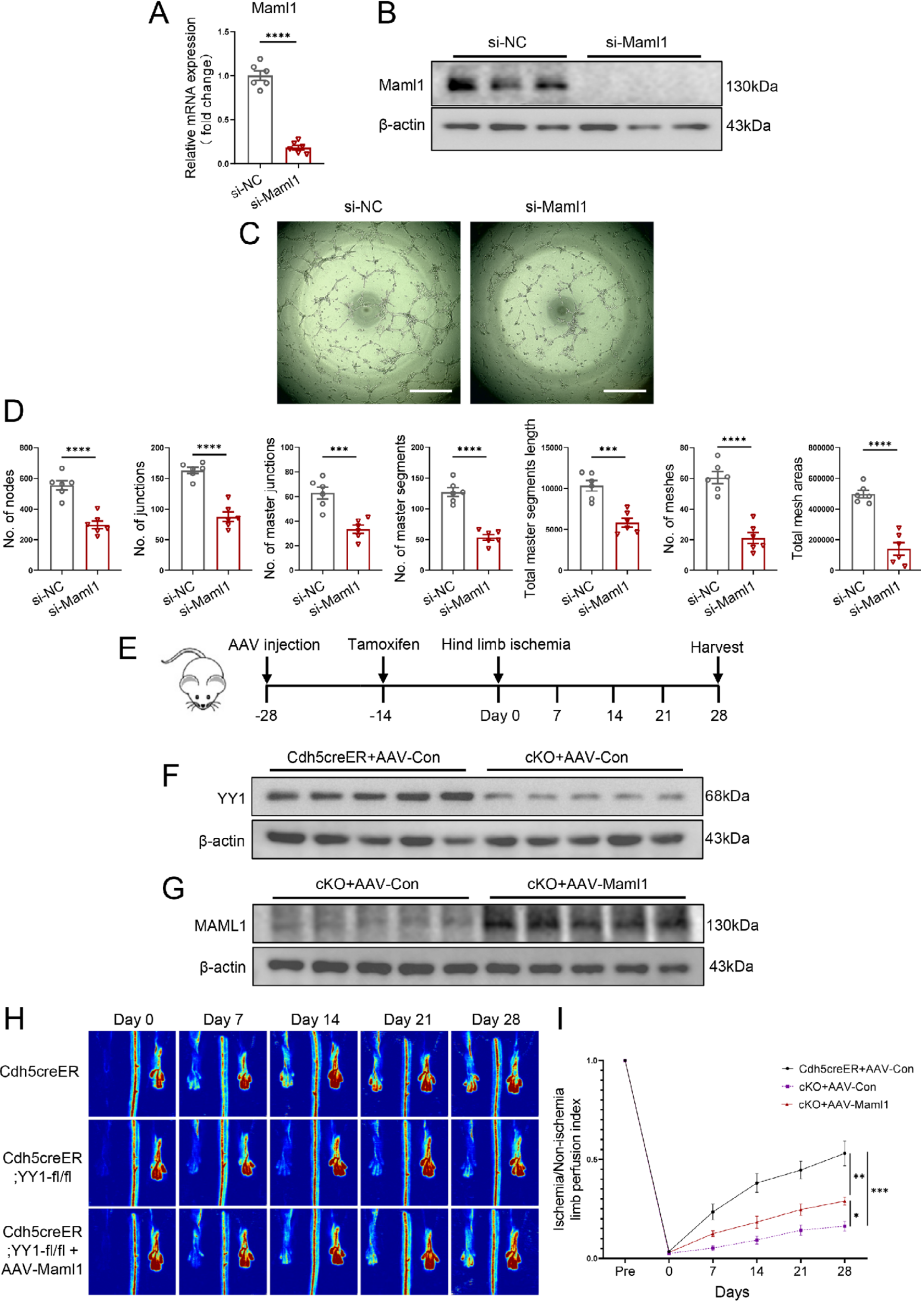
Endothelial MAML1 is required for angiogenesis and mediates YY1‐dependent vascular regeneration in vivo. (A) Relative mRNA expression level of Maml1 in hESC‐ECs transfected with si‐NC or si‐Maml1 for 48 h. ***p < 0.001, (n = 6). (B) Western blot analysis of MAML1 protein expression in hESC‐ECs treated as in (A). (C) Representative microscopic images of tube formation assay using hESC‐ECs transfected with si‐NC or si‐Maml1. Scale bar: 1 mm. (D) Quantitative analysis of the tube formation assay shown in (C), including the number of nodes, junctions, segments, and total mesh area etc. ***p < 0.001, ****p < 0.0001. (n = 6). (E) Schematic timeline of the in vivo rescue experiment. Endothelial‐specific YY1 knockout mice (*Cdh5‐CreER;Yy1^fl/fl^*, cKO) and control mice (Cdh5creER) were injected with AAV9 vectors expressing either Maml1(AAV‐Maml1) or control vector (AAV‐Con). Tamoxifen was administered to induce YY1 knockout, followed by hindlimb ischemia surgery. (F) Western blot analysis showing YY1 protein expression in hind limb muscle ECs isolated from the indicated groups at day 28 post‐ischemia. (G) Western blot analysis showing MAML1 protein expression in hind limb muscle ECs isolated from the indicated groups at day 28 post‐ischemia. (H) Representative laser Doppler perfusion images of hindlimbs at the indicated time points after ischemia. (I) Quantification of hindlimb blood perfusion over time: *Cdh5‐CreER*+AAV‐control vector, *Cdh5‐CreER;Yy1^fl/fl^* +AAV‐control vector and *Cdh5‐CreER;Yy1^fl/fl^* +AAV‐Maml1 vector. *p < 0.05, **p < 0.01, ***p < 0.001, ****p < 0.0001. (n = 5). Data are presented as mean ± SEM. Statistical significance was determined by unpaired two‐tailed *t*‐test (A, D) or two‐way ANOVA with *Tukey's* post‐hoc test (I).

To further establish MAML1's pro‐angiogenic role, siRNA‐mediated knockdown of *Maml1* (Figure 5A,B) impaired endothelial tube formation (Figure 5C,D). Because MAML1 acts downstream of YY1 in vivo, we performed a rescue experiment: AAV‐MAML1 was delivered 4 weeks before ischemia to allow overexpression, and hindlimb ischemia was induced 2 weeks after Tam (Figure 5E). At 4 weeks after ischemia, ECs isolated from limb muscle showed significantly higher MAML1 protein in AAV‐MAML1‐treated mice than in controls (Figure 5F,G), and MAML1 overexpression in *Cdh5‐CreER;Yy1‐fl/fl* mice significantly improved reperfusion (Figure 5H,I). Altogether, these results indicate that ischemia‐induced YY1 promotes vascular regeneration by activating *Maml1* transcription through H3K4 trimethylation.

### 2.5 CD4^+^ Tregs Promote Vascular Regeneration Through Activating Endothelial YY1/MAML1 in a Paracrine Manner

**FIGURE 6:**
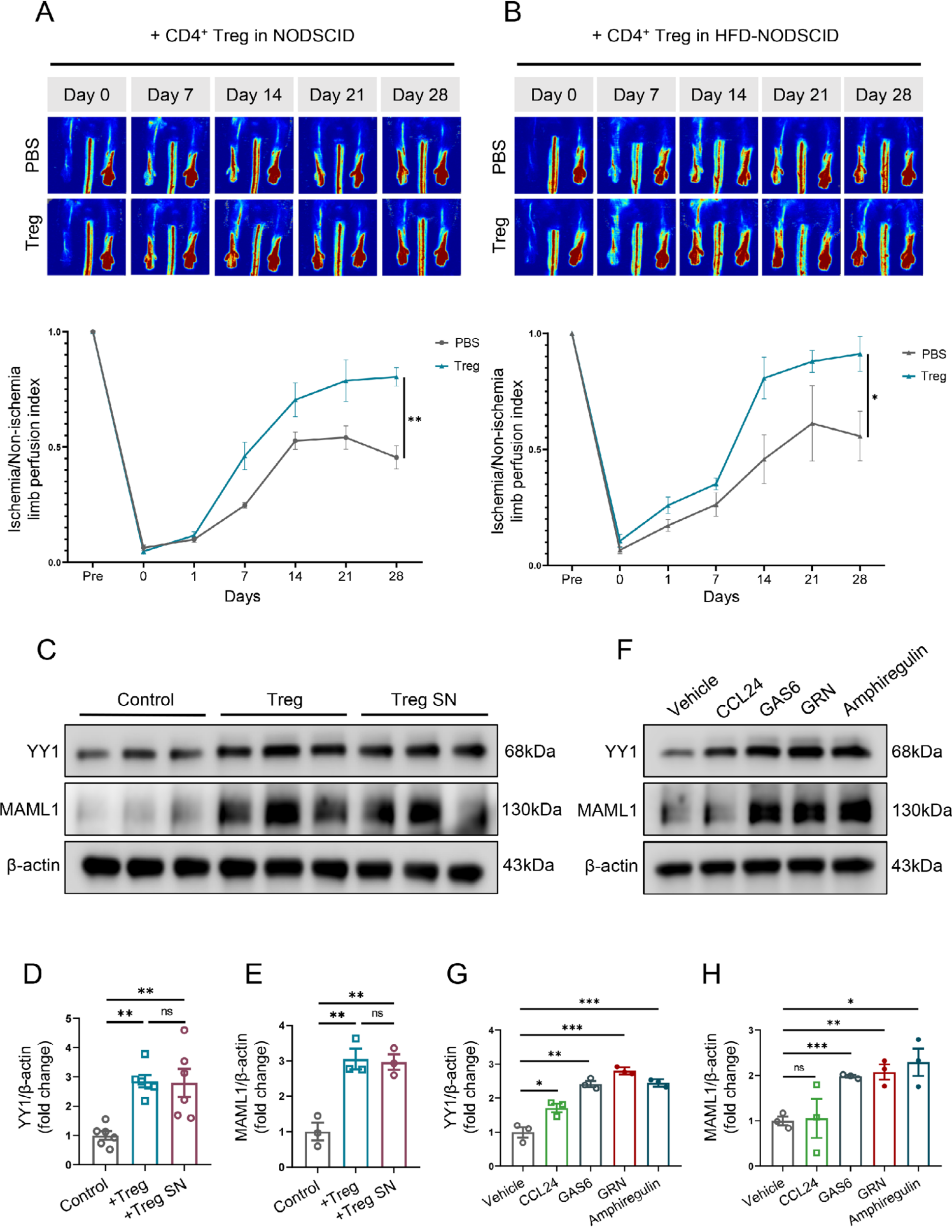
CD4^+^ Tregs promote vascular regeneration in a paracrine manner (A, B) Representative Laser Doppler perfusion imaging and quantification of hindlimb blood perfusion in NOD‐SCID (A) and HFD‐NOD‐SCID (B) mice treated with PBS or CD4^+^ Treg (5 × 10^6^ CD4^+^ Tregs per mouse). *p < 0.05, **p < 0.01. (n = 5–6). (C‐E) Western blot (C) and quantitative analysis (D, E) of YY1, MAML1, and β‐actin in Control, CD4^+^ Treg, and Treg supernatant (Treg SN) groups. Band intensities are normalized to β‐actin. **p < 0.01, (ns, no significant difference). (n = 3–6). (F–H) Western blot (F) and quantitative analysis (G, H) of YY1 and MAML1 in Vehicle, CCL24, GAS6, GRN, or Amphiregulin‐treated cells. *p < 0.05, **p < 0.01, ***p < 0.001. (n = 3). Data are presented as mean ± S.E.M. . Each dot represents 1 biological replicate. The *p* values were calculated by unpaired two‐tailed *t*‐test (A, B) or two‐way ANOVA with *Tukey's* multiple comparisons test (D, E, G, H).

We sought to identify the upstream cellular mediator of YY1 during sprouting angiogenesis after ischemic injury. Given that endothelial‐specific *Yy1* ablation markedly increased inflammatory responses (Figure 2F), we hypothesized that YY1 upregulation after ischemia is regulated by immune suppressors. CD4^+^ Tregs, being master regulators of immune homeostasis, have been implicated in tissue regeneration (for review, see [20, 21]), including promotion of cardiomyocyte regeneration after neonatal heart injury [22, 23]. To test whether CD4^+^ Tregs functionally enhance post‐ischemic vascular repair, we purified CD4^+^ Tregs from *NOD.Foxp3^hCD2^* mice, in which surface hCD2 is driven by the *Foxp3* promoter, enabling purification with an anti‐hCD2 antibody. hCD2^+^ Tregs were purified from the spleen and adoptively transferred into immunodeficient *NOD.SCID* mice after femoral artery ligation. Weekly laser Doppler imaging over 4 weeks revealed a significant improvement in blood reperfusion after Treg treatment (Figure 6A). To determine whether CD4^+^ Tregs also promote vascular regeneration in T2D, we fed NOD.SCID mice a high‐fat diet for 3 months, induced hindlimb ischemia, and adoptively transferred hCD2^+^ Tregs. High‐fat‐fed NOD.SCID mice showed increased body size and weight (Figure S6A,B), as well as elevated fasting blood glucose (Figure S6C). Weekly laser Doppler imaging over 4 weeks demonstrated that Treg transfer significantly improved reperfusion (Figure 6B), highlighting the therapeutic potential of CD4^+^ Treg for diabetic PAD.

**FIGURE 7:**
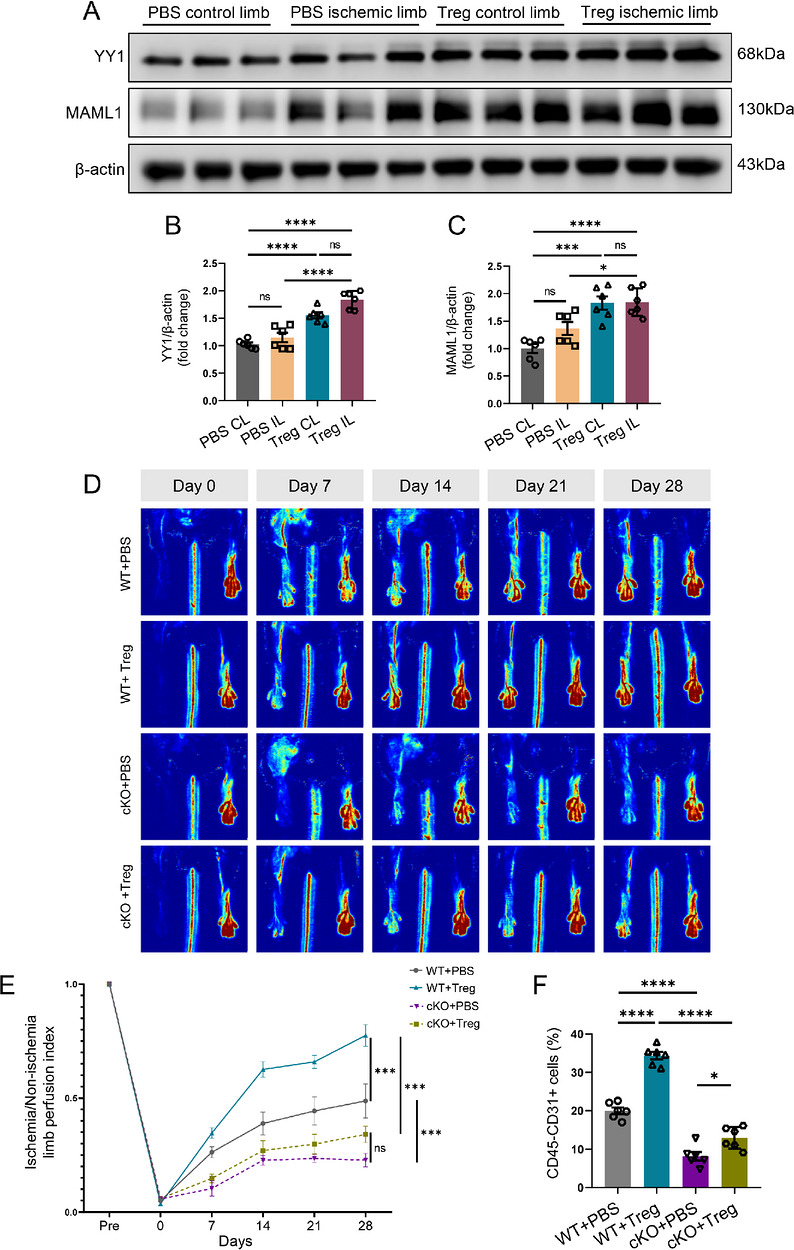
CD4^+^ Tregs promote vascular regeneration through activating endothelial YY1/MAML1 (A) Western blot of YY1, MAML1, and β‐actin in ischemic (IL) and non‐ischemic (CL) limbs of PBS or CD4^+^ Treg‐treated *Cdh5‐CreERT* (WT) and *Cdh5‐CreERT;Yy1^fl/fl^* (cKO) mice. with β‐actin as an internal control. (B‐C) Quantitative analysis of YY1/β‐actin (B) and MAML1/β‐actin (C) ratios from (A). *p < 0.05, ***p < 0.001, ****p < 0.0001, (ns, no significant difference). (n = 6). (D) Representative Laser Doppler perfusion imaging and quantification of hindlimb blood perfusion in WT+PBS, WT+Treg, cKO+PBS, and cKO+Treg groups over 28 days. (E) Quantification of hindlimb blood perfusion ratio of (D). ***p < 0.001, (ns, no significant difference). (n = 6). (F) Flow cytometry analysis of CD45^−^CD31^+^ endothelial cells in ischemic limbs. Percentage of positive cells is shown. *p < 0.05, ****p < 0.0001. (n = 6). Data are presented as mean ± S.E.M. . Each dot represents 1 biological replicate. The *p* values were calculated by two‐way ANOVA with *Tukey's* multiple comparisons test (B, C, E, F).

We next asked whether CD4^+^ Tregs modulate endothelial YY1/MAML1 expression. Murine lung ECs were cocultured with activated hCD2^+^ Tregs (3:1 ratio) purified from *NOD.Foxp3^hCD2^* mice or with Treg culture supernatant (SN) for 48 h. Both Tregs and Treg SN significantly increased endothelial YY1 and MAML1 expression (Figure 6C–E). Our previous scRNA‐seq analysis identified several CD4^+^ Treg‐derived paracrine factors, including, *Ccl24*, *Gas6*, *Grn* and *Areg*, which are upregulated during neonatal heart regeneration [22, 23]. To examine whether these ligands drive endothelial YY1/MAML1, we treated lung ECs with CCL24 (50 ng/mL), GAS6 (100 ng/mL), GRN (1 ug/mL), and AREG (100 ng/mL) for 48 h. All four factors significantly increased YY1 expression, while GAS6, GRN, and AREG also significantly elevated MAML1 expression (Figure 6F–H). In vivo, hCD2^+^ Tregs were significantly enriched in the spleen and ischemic muscle 4 weeks after ischemic injury (Figure S7A,B). To determine whether these infiltrated Tregs regulate endothelial YY1/MAML1 expression, we performed Western blot on CD31^+^ ECs purified from non‐ischemic and ischemic limbs at 4 weeks after injury. Adoptive transfer of hCD2^+^ Tregs significantly increased YY1 and MAML1 expression in ECs from both tissues (Figure 7A–C). We further tested causality by depleting Tregs using lytic anti‐hCD2 antibodies in *NOD.Foxp3^hCD2^* mice, in which Tregs express surface human CD2 (Figure S8A‐E). Treg depletion impaired ischemic reperfusion (Figure S8F,G), and ECs isolated from these mice showed reduced YY1 expression (Figure S8H) along with decreased expression of *Ccl24*, *Gas6*, *Grn*, and *Areg* (Figure S8I). Under diabetic conditions, db/db mice exhibited markedly impaired post‐ischemic reperfusion (Figure S9A‐C), with endothelial downregulation of *Ccl24*, *Gas6*, *Grn*, and *Areg* (Figure S9D), and reduced YY1 occupancy at the *Maml1* promoter (Figure S9E). Because hyperglycemia might directly affect YY1, we treated hESC‐ECs with 25 mM D‐glucose with 25 mM L‐glucose as an osmotic control [4], and observed no change in YY1 expression or YY1 enrichment at the *Maml1* promoter (Figure S9F,G). However, expression of *Ccl24*, *Gas6*, *Grn*, and *Areg* was downregulated in activated hCD2^+^ Tregs exposed to D‐glucose (Figure S9H). To assess whether this reflected impaired Treg survival rather than altered secretory function, we quantified apoptosis by Annexin V/propidium iodide (PI) staining. D‐glucose did not change the percentage of Annexin V^+^PI^−^ Tregs relative to L‐glucose controls (Figure S9I). These results suggest that hyperglycemia diminishes the ability of Tregs to secrete these trophic factors rather than affecting Treg survival. Given that GAS6, GRN, and AREG increase MAML1 expression in ECs (Figure 6F, H), we assessed their receptors after ischemia and found marked endothelial upregulation of *Axl* (Gas6 receptor), *Epha2* (Grn receptor) and *Egfr* (Areg receptor) (Figure S9J, K). Each ligand increased YY1 and MAML1, whereas knockdown of *Axl*, *Epha2* or *Egfr* abrogated these effects (Figure S10). Together, these data support a model in which post‐ischemic Tregs are necessary to induce endothelial YY1/MAML1; Treg deficiency (as in T2D) contributes to inadequate YY1 upregulation and impaired vascular repair.

Lastly, we evaluated whether CD4^+^ Tregs promote vascular regeneration through activating endothelial YY1. CD4^+^CD25^+^ Tregs were purified from *Yy1^fl/fl^* spleens and adoptively transferred into *Cdh5‐CreERT;Yy1^fl/fl^* (cKO) or *Cdh5‐CreERT* (WT) mice. Two weeks after the last dose of tamoxifen, mice underwent femoral artery ligation followed by Treg transfer. Weekly laser Doppler imaging over 4 weeks revealed that Tregs significantly improved reperfusion in controls (WT+Treg vs. WT+PBS) but not in endothelial YY1‐deficient mice (cKO+Treg vs. cKO+PBS), and the pro‐reperfusion effect of Tregs in promoting reperfusion was markedly reduced without endothelial YY1 (Figure 7D,E). Consistently, immunostaining for CD31 (Figure S11A) or flow cytometry (Figure S11B) targeting CD45^−^CD31^+^ cells revealed increased endothelial coverage in Treg‐treated controls (WT+Treg vs. WT+PBS) but not in cKO mice (cKO+Treg vs. cKO+PBS), and the action of Tregs in promoting EC growth after injury was markedly reduced without endothelial YY1 (Figure 7F). Taken together, these results suggest that Treg therapy promotes post‐ischemic vascular regeneration, including in T2D settings, and that its efficacy depends on activation of the endothelial YY1/MAML1 axis.

## 3 Discussion

**FIGURE 8:**
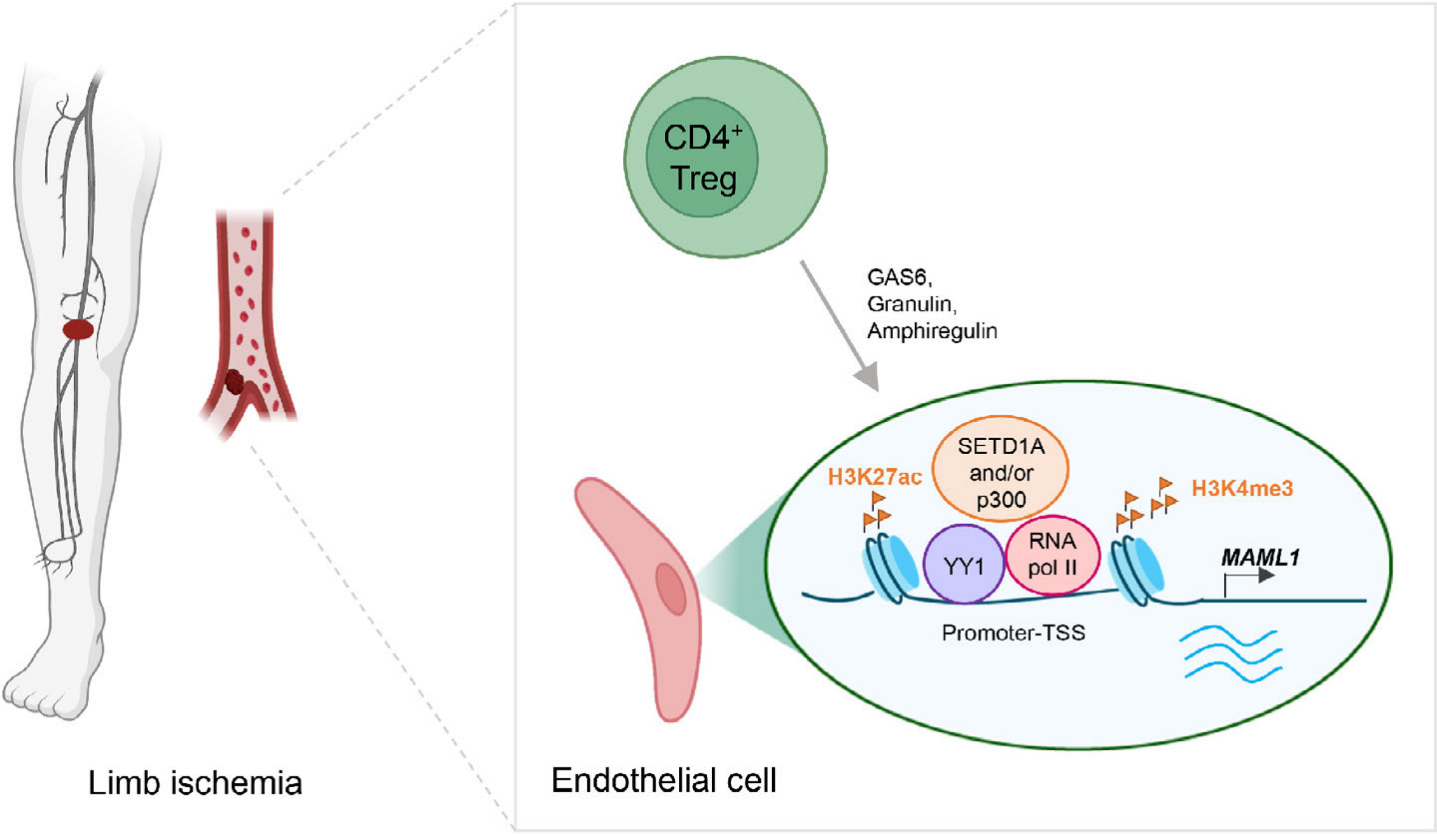
A hypothetical model of this study. In this study, we identify a CD4^+^ Treg‐based therapeutic strategy for diabetic PAD and delineate a mechanism by which these cells enhance sprouting angiogenesis. After ischemic injury, endothelial YY1 is upregulated by CD4^+^ Tregs through paracrine factors, including GAS6, granulin, and amphiregulin. YY1 activates endothelial Notch signaling by promoting transcription of the Notch coactivator *MAML1* through epigenetic mechanisms, recruiting the H3K4me3 writer such as SETD1A and the H3K27ac writer p300 to deposit activating histone marks and enhance *MAML1* expression.

Immune activation is among the earliest responses to tissue injury, as leukocytes migrate to the wound and release cytokines, chemokines, metabolites, and growth factors. These paracrine signals drive key biological processes to support tissue repair, including host defense, clearance of necrotic tissues, angiogenesis, and progenitor cell proliferation [24]. Although ischemic injury can promote endothelial repair, our previous work showed that CD4^+^ Th1 and CD8^+^ T cells suppress sprouting angiogenesis after ischemia, especially in diabetes [4, 5]. Both effector T cell‐subsets are likely restrained by CD4^+^ Tregs, master regulators of immune homeostasis [20, 21]. We previously found that CD4^+^FOXP3^+^ Tregs increase after ischemia, but this response is lost in T2D [4]; how CD4^+^ Tregs promote sprouting angiogenesis, however, has remained unclear. Here, we identify a CD4^+^ Treg‐based therapeutic strategy for diabetic PAD and delineate a mechanism by which these cells enhance sprouting angiogenesis (Figure 8). YY1 is upregulated in ECs after ischemia, but this induction is blunted in T2D; CD4^+^ Tregs restore endothelial YY1 through paracrine factors, including GAS6, granulin, and amphiregulin. Consistent with a functional requirement of endothelial YY1, CD4^+^ Tregs fail to improve post‐ischemic reperfusion and angiogenesis in endothelial‐specific *Yy1* knockout mice, and *Yy1* depletion increases inflammatory gene expression in ECs, indicating that YY1 limits vascular inflammation and is positively regulated by immunosuppressive CD4^+^ Tregs. Mechanistically, YY1 activates endothelial Notch signaling by transcriptionally upregulating the Notch coactivator MAML1. YY1 recruits the H3K4me3 writer such as SETD1A and the H3K27ac writer p300 to deposit activating histone marks at the *MAML1* promoter, thereby enhancing its expression. Consistent with this pathway, AAV‐mediated *MAML1* overexpression improved post‐ischemic reperfusion in YY1 deficient mice. Collectively, endothelial YY1 activation drives Notch signaling to promote sprouting angiogenesis after ischemia; this pathway is impaired in diabetic PAD but can be rescued by adoptive transfer of CD4^+^ Tregs.

During embryonic retinal development, YY1 has been reported to promote sprouting angiogenesis by repressing Notch signaling [10]. EC‐specific *Yy1* deletion leads to embryonic lethality with defective angiogenesis and vascular maturation, accompanied by increased *Hey1* expression [15]. Mechanistically, in HUVECs, YY1 was shown to bind RBPJ and disrupt assembly of the NICD‐MAML1‐RBPJ activation complex required for Notch target gene induction. Notch signaling is a well established regulator of vascular development and homeostasis, controlling endothelial proliferation, migration, angiogenesis, and arterial‐venous specification [25]. In contrast, in our hESC‐EC system we did not detect YY1‐RBPJ interaction by co‐IP, and YY1 loss reduced, rather than increased, *Hey1* expression in E11.5 embryonic ECs and in adult ECs from ischemic muscle. Instead, our data indicate that YY1 directly activates the Notch pathway upstream of target‐gene output by binding and transcriptionally activating MAML1. In hESC‐ECs, YY1 promoted *MAML1* transcription as demonstrated by luciferase reporter assays, and physically associated with MAML1 as evidenced by co‐IP. Given YY1's ability to recruit chromatin coactivators that install activating histone marks, YY1 likely facilitates Notch activation by enhancing chromatin accessibility and coactivator occupancy at Notch regulatory genes. YY1 is a context‐dependent transcriptional regulator whose activating or repressive functions are shaped by cellular environment, cofactors, and locus specificity (for review, see [11]). Unlike homeostatic conditions, the post‐ischemic endothelium experiences hypoxia, inflammation, oxidative stress, and altered signaling crosstalk, which can reprogram YY1's regulatory logic. Consistent with this, we observed distinct YY1 target genes in non‐ischemic versus ischemic limbs. Regarding repression, genes bound by YY1 were significantly upregulated after endothelial YY1 knockout and were enriched for inflammatory pathways, indicating that endothelial YY1 normally represses inflammation after ischemia. Together, these findings support a model in which YY1 acts both an activator (for Notch signaling) and a repressor (for inflammatory signaling). Its upregulation after ischemia may protect endothelium by simultaneously promoting angiogenesis and repressing inflammation – a protective response that is lost in diabetic ECs due to insufficient Tregs.

Taking MAML1 as an example, we delineate an epigenetic mechanism by which YY1 regulates Notch regulator transcription in ECs. Multiple lines of evidence support a model in which YY1 functions as a scaffold that recruits H3K4 methyltransferase and H3K27 acetyltransferase machineries to the *MAML1* promoter. Co‐IP in hESC‐ECs showed that YY1 interacts with SETD1A, MLL1, MLL2, and the shared COMPASS/MLL core subunit WDR5. YY1 also associated with p300 and RNA pol II in hESC‐ECs and murine primary ECs. These interactions are consistent with YY1 forming or stabilizing assemblies that bring COMPASS/MLL family H3K4 methyltransferases including SETD1A, MLL1 and MLL2, and the H3K27 acetyltransferase p300 to active promoters, resulting in co‐occurrence of H3K4me3 and H3K27ac at *MAML1*. YY1's interaction with RNA pol II suggests a role in transcription initiation and/or early elongation. Notably, the N‐terminal domain of SETD1A can bind RNA pol II [26], and p300/CBP has been shown to interact with RNA pol II [27]. However, whether these factors are co‐recruited to chromatin simultaneously or in a defined temporal order remains to be determined. ChIP‐seq in human or CUT&RUN‐seq in murine ECs further revealed overlapping YY1, H3K4me3 and H3K27ac peaks at the*MAML1* promoter. *YY1* knockdown in hESC‐ECs reduced H3K4me3, H3K27ac, SETD1A and p300 at the *MAML1* promoter and decreased *MAML1* mRNA, highlighting the importance of these marks for *MAML1* transcription. Consistently, *SETD1A* knockdown also diminished *MAML1* transcription. Collectively, our data support a model in which YY1 helps recruit and/or stabilize COMPASS/MLL complexes and p300 at the *MAML1* promoter in ECs, thereby coupling promoter‐proximal H3K4 trimethylation and H3K27 acetylation with productive transcription by RNA pol II. The precise composition and simultaneity of these assemblies with WSC‐p300‐RNA pol II at the *MAML1* locus remain to be established. We hypothesize that loss of YY1 destabilizes these coactivator‐RNA pol II interactions at the *MAML1* promoter, leading to reduced promoter‐proximal methylation and acetylation and consequently less efficient transcription.

Further studies are needed to determine how Tregs sense self‐antigens (and which antigens are involved) to trigger paracrine factor release, and to elucidate how these factors regulate YY1/MAML1 by specifically deleting each cognate receptor in endothelial cells in vivo. Another limitation is our predominant reliance on male mice, which constrains generalizability. Estrogen can expand CD4^+^ Tregs [28], potentially complicating direct sex comparisons. Future experiments should include both sexes to dissect hormone‐dependent effects. In summary, ischemia‐induced, CD4^+^ Treg‐supported upregulation of endothelial YY1 restores Notch competence by epigenetically activating MAML1 and promoting sprouting angiogenesis. This CD4^+^ Treg‐YY1‐MAML1 axis is dysregulated in diabetes, contributing to impaired vascular repair. We further demonstrated that diabetic PAD can be rescued by adoptive transfer of CD4^+^ Tregs or potentially by therapeutically enhancing YY1 function or strengthening its chromatin coactivator partnerships. Thus, YY1 emerges as a novel regulator linking immune cues to endothelial chromatin state and Notch‐driven angiogenic programs, nominating it as a tractable target for treating diabetic PAD.

## 4 Experimental Section

### 4.1 Mice

Animal care and use were approved by the Animal Experimentation Ethics Committee of CUHK (19‐212‐MIS, 20‐083‐MIS, 21‐197‐MIS and Shenzhen Glorybay Biotech Co., Ltd. (RW‐IACUC‐25‐0110, RW‐IACUC‐25‐0111, RW‐IACUC‐25‐0112, RW‐IACUC‐25‐0113). All procedures were conducted in accordance with institutional guidelines. Male mice were housed at CUHK or Shenzhen Glorybay Biotech Co., Ltd. under a 12‐h light‐dark cycle and maintained on a standard chow diet. Mouse strains used as previously described: *Cdh5‐*Cre [29], *Cdh5‐*CreERT [4], *YY1^fl/fl^* [6, 7], *NOD.Foxp3^hCD2^* [22], *Lepr^db/+^* [4, 5], *Lepr^db/db^* [4, 5], and *NOD.SCID*. T2D was induced by feeding a high‐fat diet (Research Diets, Cat No. D12492) for 3 months; mice with fasting blood glucose > 11.1 mmol/L were classified as diabetic and enrolled. For *Cre* activation, tamoxifen (MedChemExpress, Cat No. HY‐13757A) was prepared in corn oil (10 mg/mL) and administered intraperitoneally (i.p.) at 100 ug/g once daily for 5 days in 6–8 week‐old mice. Surgeries were performed 2 weeks after tamoxifen washout. CD4^+^ Tregs were depleted in *NOD.Foxp3^hCD2^* mice using 0.25 mg of the lytic anti‐human CD2 antibody (clone YTH655) injected intravenously (i.v.) daily for 7 days as previously described [30]. To overexpress MAML1 in ECs, an AAV9 vector encoding *Maml1* under a *Tie2* promoter was administered intravenously at 200ul of 1*10^12 vg/mL per mouse. Euthanasia was performed under 2.5% isoflurane anesthesia followed by cervical dislocation. All animals were analyzed unless they died of non‐procedure‐related causes before the predefined endpoint.

### 4.2 Induction of Hindlimb Ischemia

Hindlimb ischemia was performed as described previously [31]. After induction of anaesthesia with isoflurane, the surgical site was shaved and disinfected. The left femoral artery was isolated and ligated with 6‐0 surgical silk. Blood perfusion was measured by a Laser Doppler Imager (Moor Instruments, Cat. No. moorLDI‐2). During scanning, anesthetized mice were placed on a heating pad set to 37°C to minimize temperature‐related variability. Perfusion was expressed as the ratio of blood flow in the ischemic to the contralateral non‐ischemic hindlimb. At study endpoints, mice were euthanized under isoflurane anesthesia followed by cervical dislocation. Gastrocnemius and adductor muscles from both legs were harvested for subsequent analyses.

### 4.3 In Vivo EdU Incorporation Assay

Mice received 10 mM EdU (Invitrogen, Cat. No. C10632) by i.p. injection at a dose of 70 µL per mouse. Gastrocnemius and adductor muscles were harvested 24 h later. For flow cytometric quantification, muscles were enzymatically dissociated as described below, and resulting cells were fixed, permeabilized, and incubated with the EdU reaction cocktail for 30 min at room temperature per the manufacturer's instructions. EdU‐positive cells were analyzed on a CytoFLEX flow cytometer (Beckman Coulter), and data were processed in FlowJo.

### 4.4 Human Embryonic Stem Cell and hESC‐ECs Cultures

Human embryonic stem cell (hESC) line H9 (Wicell, Madison, WI) was maintained in mTeSR Plus medium (Stemcell Technologies) per manufacturer's instructions, with daily medium changes to preserve pluripotency, and passaged using Accutase (Life Technologies). For endothelial differentiation, we adopted our reported protocol [32]. Briefly, hESCs were plated on growth factor‐reduced Matrigel‐coated plates at 37 000–47 000 cells/cm^2^ and cultured overnight in mTeSR supplemented with the ROCK inhibitor Y‐27632 (10 µM). The next day, medium was replaced N2B27 (1:1 DMEM/F12:Neurobasal with Glutamax, β‐Mercaptoethanol, and N2/B27; all Life Technologies) containing CHIR‐99021 (6‐8 µM) and hBMP4 (25 ng/mL) for 3 days. On day 4, cells were refreshed with Stem Pro‐34 SFM (Gibco) supplemented with VEGF‐A (200 ng/mL, Peprotech) and Forskolin (2 µM/mL, Peprotech) for 2 days with daily changes. On day 6, CD144^+^ cells were enriched using human CD144 (VE‐Cadherin) MicroBeads (Miltenyi Biotec), seeded onto collagen‐coated dishes at 3.7 × 10^4^ cells/cm^2^, and cultured in Endothelial Cell Growth Medium‐2 (Lonza) for 2 days before use. For siRNA transfection, hESC‐ECs were seeded in 6‐well plates at 50–60% confluency 24 h prior to transfection. siRNA complexes were prepared by mixing siRNA (final concentration: 20 nM) with 50 µL Opti‐MEM Reduced Serum Medium and, separately, Lipofectamine RNAiMAX (1.5 µL, Invitrogen, 13778‐150) with 50 µL Opti‐MEM. Mixtures were incubated for 5 min, combined, and incubated for 20 min at room temperature before being added to cells for a 48 h transfection. YY1, SETD1A and MAML1 siRNAs (GenePharma biotech) were used; sequences are listed in Table S3.

### 4.5 Isolation of Mouse Tregs

For *NOD.SCID* recipients, hCD2^+^ Tregs were isolated from the spleens of *NOD.Foxp3^hCD2^* using CD2 MicroBeads (Miltenyi Biotec, 130‐091‐114) according to the manufacturer's instructions. Briefly, splenocytes were washed twice in cold, degassed buffer (PBS, 1% BSA, 2 mM EDTA) and centrifuged at 300 g for 10 min to remove platelets. Cells were resuspended at 10^7^ total cells per 80 µL buffer, incubated with CD2 MicroBeads (20 µL per 10^7^ cells) for 15 min at 4°C, washed with 2 mL buffer, centrifuged at 300 g for 10 min, and resuspended in 500 µL buffer. Positive selection was performed on an autoMACS Pro Separator (Miltenyi Biotec); the magnetically retained fraction from MS Columns contained the hCD2^+^ Tregs.

For *Cdh5‐CreERT;Yy1^fl/fl^* (cKO) and *Cdh5‐CreERT* (WT) mice, CD4^+^CD25^+^ Tregs were isolated from the spleen of *Yy1^fl/fl^* or *Yy1^fl/+^* mice using the CD4^+^CD25^+^ Regulatory T Cell Isolation Kit (Miltenyi Biotec, 130‐091‐041). Splenocytes were prepared as above and kept in prechilled buffer (PBS, 1% BSA, 2 mM EDTA). Non‐CD4^+^ cells were first depleted by incubating the suspension with the provided Biotin‐Antibody Cocktail and Anti‐Biotin MicroBeads for 15 min at 4°C, followed by magnetic separation on an LD Column in a MACS Separator; the flow‐through contained enriched CD4^+^ fraction was centrifuged, resuspended in buffer, labeled with an anti‐CD25‐PE antibody for 15 min at 4°C, then incubated with Anti‐PE MicroBeads for 15 min at 4°C. Magnetic separation was performed using two sequential MS Columns in a MACS Separator (Miltenyi Biotec). The magnetically retained fraction contained the CD4^+^CD25^+^ Tregs.

### 4.6 Adaptive Transfer of Tregs

For transfer experiments, Tregs were freshly isolated from spleens of 8 week‐old male wild type mice using either CD2 MicroBeads or the CD4^+^CD25^+^ Regulatory T Cell Isolation Kit (Miltenyi Biotec), as described above. A total of 5 × 10^6^ Tregs in 100 µL sterile PBS were administered by tail vein injection into 8‐week‐old male recipient mice 1 day after induction of hindlimb ischemia.

### 4.7 Single Cell Isolation

Single‐cell suspensions were prepared from muscle, spleen and lungs. For muscle, the injured gastrocnemius was excised immediately after euthanasia, minced on ice in a 2 mL tube, and digested in PBS containing Collagenase II (800U/mL, Worthington, LS004177) and dispase (11U/mL, Thermo Fisher Scientific, 17105041) for 40 min at 37°C with gentle shaking (100 rpm). Digestion was quenched with an equal volume of 20% FBS in HBSS, and the suspension was sequentially filtered through 70 µm (SPL, 93070) and 40 µm (SPL, 93040) strainers. For spleen, tissue was transferred to a 35 mm dish with 2 mL cold PBS, mechanistically dissociated using a 10 mL syringe plunger, and filtered through 70 and 40 µm strainers. Cells were pelleted by centrifugation at 300 g for 5 min at 4°C, resuspended in 10 mL RBC lysis buffer (Beyotime, C3702) for 5 min on ice, washed by centrifugation at 300 g for 5 min at 4°C, and resuspended in PBS before transfer to a 1.5 mL tubes for downstream flow cytometry. For lungs, tissues were aseptically removed, rinsed in PBS, minced into ∼ 1 × 2 mm^2^, and digested with collagenase II (400U/mL, Worthington, LS004177) and dispase (5.5U/mL, Thermo Fisher Scientific, 17105041) at 37°C for 45 min with agitation. Digestions were quenched with an equal volume of 20% FBS in HBSS and filtered through 70 and 40 µm strainers. The resulting single‐cell suspensions were used immediately for analysis or sorting by flow cytometry.

### 4.8 Isolation of Mouse CD31^+^ ECs

Following skeletal muscle or lung tissue digestion, CD31^+^ ECs were isolated using CD31 MicroBeads (Miltenyi Biotec, 130‐097‐418) per the manufacturer's instructions. Briefly, cells were washed twice in cold, degassed buffer (PBS, 1% BSA, 2 mM EDTA) and centrifuged at 300 g for 10 min to remove platelets. Up to 10^7^ cells were resuspended in 90 µL cold buffer, incubated with CD31 MicroBeads (10 µL per 10^7^ cells) for 15 min at 4°C, washed with 2 mL buffer, and resuspended in 500 µL buffer. Positive separation was performed on an autoMACS Pro Separator (Miltenyi Biotec); the magnetically retained fraction on MS Columns contained the CD31^+^ ECs.

### 4.9 Treg and EC Cocultures

Treg‐EC coculture experiments were performed as previously described [22]. Naïve Tregs were purified from the spleens of *NOD.Foxp3^hCD2^* mice using anti‐human CD2 magnetic beads (Miltenyi Biotech, Cat. No. 130‐091‐114) following the manufacturer's instructions. For activation, 6‐well plates were coated overnight at 4°C with anti‐CD3 (10 ug/mL, Biolegend, Cat. No. 100314) and anti‐CD28 (1 ug/mL, Biolegend, Cat. No. 102112), then washed twice with PBS. Tregs were cultured for 3 days at 37°C in RPMI1640 (Gibco, Cat. No. 11875‐093) supplemented with 10% heat‐inactivated FBS (Gibco, Cat. No. A3381901), 1% sodium pyruvate (Life Technologies, Cat. No. 11360‐070), HEPES (10 mM, Thermo Fisher, Cat. No. 15630080), 2‐mercaptoethanol (50 uM, Life Technologies, Cat. No. 31350‐010), IL‐2 (40 ng/mL, Peprotech, Cat. No. 212‐12), and TGFβ (10 ng/mL, R&D systems, Cat. No. 7666‐MB‐005). ECs were then cocultured with the stimulated Tregs at a 3:1 ratio (ECs: Tregs), or exposed to Tregs‐conditioned medium (supernatant: complete medium = 1:1), or treated with individual recombinant factors: murine amphiregulin (100 ng/mL, AREG, R&D systems, Cat. No. 989‐AR‐100), chemokine ligand 24 (50 ng/mL, CCL24, Biolegend, Cat. No. 585102), granulin (1 ug/mL, GRN, Lifespan biosciences, Cat. No. LS‐G3786‐10), or growth arrest specific 6 (100 ng/mL, GAS6, R&D systems, Cat. No. 8310‐GS‐050) for 48 h at 37°C before analysis.

### 4.10 Mouse Lung CD31^+^ ECs Culture and si‐RNA Transfection

Mouse lungs were digested and CD31^+^ cells were isolated using CD31‐conjugated beads as described above. CD31^+^ ECs were seeded onto collagen‐coated dishes and cultured in Endothelial Cell Growth Medium‐2 (Lonza) for 3 days and change medium every day before use. For siRNA transfection, CD31^+^ ECs were seeded in 6‐well plates at 50–60% confluency 24 h prior to transfection. siRNA complexes were prepared by mixing siRNA (final concentration: 50 nM) with 50 µL Opti‐MEM Reduced Serum Medium and, separately, Lipofectamine RNAiMAX (2 µL, Invitrogen, 13778‐150) with 50 µL Opti‐MEM. Mixtures were incubated for 5 min, combined, and incubated for 20 min at room temperature before being added to cells for a 48 h transfection. Axl1, Epha2 and Egfr siRNAs (GenePharma biotech) were used; sequences are listed in Table S3.

### 4.11 Flow Cytometry

Single‐cell suspensions from spleen and muscle were prepared as described above. Prior to surface marker staining, Fc gamma receptors were blocked with TruStain FcX PLUS (1:50 dilution, anti‐mouse CD16/32; Biolegend, 156604) for 15 min at 4°C. Cells were then incubated with the appropriate fluorophore‐conjugated antibodies for 30 min at 4°C, washed three times with PBS containing 2% FBS, and analyzed on a CytoFLEX flow cytometer (Beckman Coulter). Dead cells were excluded using DAPI (Invitrogen, D3571) or propidium iodide (PI, BD Pharmingen), and data were analyzed with FlowJo software. Antibodies used in this study are listed in Table S4.

### 4.12 Immunofluorescence

Muscles were fixed by 4% paraformaldehyde (PFA) at 4°C overnight, washed several times in 1× PBS, and dehydrated in 30% sucrose at 4°C until the tissues sank. Samples were embedded in Optimum Cutting Temperature (OCT) compound (Sakura, 4583), frozen at −80°C, equilibrated in the cryostat (Thermo fisher, CryoStar NX70) for 20–30 min, and sectioned at 7 µm. Before staining, slides were washed in 1× PBS to remove OCT, permeabilized in 1× PBS with 0.3% Triton X‐100 (RPI, T18000‐0.05) for 10–15 min at room temperature, and blocked for 30–60 min at room temperature in 1× PBST (1× PBS + 0.1% Tween 20) containing 1% BSA (Beyotime, ST023), 22.52 mg/mL glycine (Invitrogen, 15527013) and 5% goat serum (Sigma‐Aldrich, G9023). After blocking, sections were incubated with primary antibodies diluted in 1% BSA in 1× PBST overnight at 4°C in the dark, washed 3–5 times with 1× PBST, and incubated with fluorochrome‐conjugated secondary antibodies diluted in 1% BSA in 1× PBST for 30–45 min at room temperature in the dark. After 3–5 additional PBST washes, nuclei were counterstained with DAPI (0.1‐1 µg/mL, Invitrogen, D3571) for 5–10 min at room temperature in the dark. Slides were then washed 3–5 times in 1× PBST, mounted with mounting medium (Abcam, ab104135), and imaged on a fluorescence microscope (Leica). Antibodies used are listed in Table S5.

### 4.13 Western Blot

Single‐cell suspensions from muscle were prepared as described above. Cells were lysed in 200 µL RIPA buffer on ice for 30 min, and lysates were harvested by centrifugation at 13 200 g for 3 min at 4°C. Supernatants were mixed with loading buffer (Beyotime) and heated at 95°C for 8 min. Equal amounts of protein were resolved by SDS‐PAGE and transferred to PVDF membranes (Roche). Membranes were blocked in 5% BSA in TBST for 1 h at room temperature, then incubated with primary antibodies overnight at 4°C. Horseradish peroxidase (HRP)‐conjugated anti‐mouse or anti‐rabbit secondary antibodies (Santa Cruz,1:5000) were used for detection, and 𝛽‐actin served as an internal control. Primary antibodies used are listed in Table S6.

### 4.14 Co‐Immunoprecipitation

For co‐IP, single‐cell suspensions from muscle were prepared as described above. Cells were lysed in RIPA buffer (Beyotime) supplemented with protease inhibitor (Beyotime) and dithiothreitol (DTT). Mouse tissues were minced, ground in liquid nitrogen with 200 µL IP buffer, and incubated on ice for 30 min. Lysates were harvested by centrifugation at 13 200 g at 4°C for 3 min. After precleaning, samples were incubated with antibodies overnight at 4°C with rotation, followed by incubation with Protein A/G magnetic beads (ThermoFisher) for 2 h at 4°C with rotation. Immune complexes were washed three times with RIPA buffer (5 min/wash), eluted in SDS‐PAGE loading buffer (Beyotime) at 95°C for 8 min, and analyzed by Western blot. Antibodies used are also listed in Table S7.

### 4.15 Tube Formation Assay

A 96‐well plate was coated with Matrigel (50 µL per well, Corning, 354234), left at 4°C for 10 min to flatten the surface, and then incubated at 37°C for 30 min to solidify. hESC‐ECs were seeded at 2.0×10^4^ cells per well onto the Matrigel and cultured for 6 h. Tube formation was quantified in ImageJ by measuring mean mesh size, total mesh area, number of segments, total segments length, number of nodes, and number of junctions per well.

### 4.16 Dual‐Luciferase Reporter Assay

Promoter activities were measured using the Dual‐Luciferase Reporter Assay System (Promega). hESC‐ECs were transfected in 24‐well plates with 500 ng of either MAML1 wildtype (WT) or MAML1 deletion site 1 (Del1/ ΔSite1) and site 1 (Del2/ ΔSite2) luciferase reporter constructs (deletions removing YY1‐binding motifs CCAT/ATGG), along with pRL‐TK (Promega) and YY1 modified mRNA (YY1‐modRNA). The transfection groups were: 1) eGFP‐modRNA + pGL3‐Basic + pRL‐TK; 2) YY1‐modRNA + pGL3‐Basic + pRL‐TK; 3) eGFP‐modRNA + WT+ pRL‐TK; 4) YY1‐modRNA + WT + pRL‐TK; 5) eGFP‐modRNA + Del1 (ΔSite1) + pRL‐TK; 6) YY1‐modRNA + Del1 (ΔSite1) + pRL‐TK; 7) eGFP‐modRNA + Del2 (ΔSite2) + pRL‐TK; 8) YY1‐modRNA + Del2 (ΔSite2) + pRL‐TK. At 48 h post‐transfection, cells were lysed and collected by centrifugation at 10 000 g for 5 min. Supernatants (50 µL per sample) were assayed following the manufacturer's instructions. Firefly luciferase activity was normalized to luciferase from the pRL‐TK control.

### 4.17 Modified mRNA Synthesis and Transfection

In some experiments, eGFP and YY1 modified mRNAs (modRNA) were synthesized as previously described [33]. Briefly, open reading frames were PCR‐amplified from plasmids encoding eGFP or human YY1. In vitro transcription was performed using the MEGAscript T7 kit (Thermofisher, Cat. No. AMB13345) with a custom ribonucleoside blend containing 3′‐O‐Me‐m7G(5’)ppp(5’)G cap analog (New England Biolabs, Cat. No. S1411L), ATP and GTP (TriLink Biotechnologies, Cat. No. N‐1014‐1), and 5‐methylcytidine triphosphate and pseudouridine triphosphate (TriLink Biotechnologies, Cat. No. N‐1091‐1). For transfection, modRNA and RNAiMAX transfection reagent were each diluted separately in Opti‐MEM (Invitrogen), combined, and incubated for 15 min at room temperature to form complexes. For in vitro transfection, 1 µg modRNA per well was added to cells seeded in six‐well plates, using either DMEM supplemented with 2% FBS and 200 ng/mL B18R (eBioscience) or in Pluriton Reprogramming Medium (Stemgent).

### 4.18 Quantitative Real Time‐PCR (RT‐qPCR)

Total RNA from tissues and cells was extracted with TRIzol reagent (Vazyme, China). cDNA was synthesized using the iScript cDNA Synthesis Kit (Bio‐Rad, USA) according to the manufacturers’ instructions. Briefly, hESC‐ECs were lysed directly in TRIzol. Tissues were minced, dissociated into single cells, and then lysed in TRIzol. RT‐qPCR was performed on a CFX Connect Read‐Time PCR Detection system (Bio‐Rad) using SYBR Green chemistry (Bio‐Rad) according to the manufacturer's protocol. Gene expression was normalized to β‐actin, and relative expression levels were calculated using the internal control for each sample. Primer sequences are provided in Table S8.

### 4.19 RNA Sequencing

Total RNA quality was assessed on an Agilent TapeStation to determine RNA integrity numbers (RINs) before library preparation. mRNA was enriched with poly‐T oligo‐conjugated magnetic beads, fragmented, and reverse‐transcribed to cDNA. During second‐strand synthesis, dUTP was incorporated so that the second strand was not amplified, enabling strand‐specific libraries. cDNA was end‐repaired, ligated to indexed adapters, and PCR‐amplified. After each step, nucleic acids were purified with AMPure XP beads (Beckman Coulter). Libraries were quantified, pooled, and sequenced as 150 bp paired‐end reads on an Illumina HiSeq X platform.

### 4.20 Cleavage Under Targets and Release Using Nuclease (CUT&RUN) Sequencing

CUT&RUN was performed on ECs using the CUT&RUN Assay Kit (Cell Signaling Technology, Cat. No. 86652) per the manufacturer's instructions. Briefly, ECs were harvested, washed, and bound to concanavalin A‐coated magnetic beads. Bead‐bound cells were permeabilized and incubated with primary antibody at 4°C overnight with gentle agitation. After washing, cells were incubated with recombinant pAG‐MNase for 1 h at 4°C. Targeted cleavage was initiated by adding calcium chloride and carried out for 30 min at 4°C. Reactions were stopped with stop buffer, and released chromatin fragments were collected from the supernatant. An input control (antibody untreated) was processed in parallel to assess background and normalize enrichment. DNA was purified using kit spin columns, and libraries were prepared with the NEBNext DNA Library Prep Kit for Illumina (New England Biolabs, Cat. No. E7645). Paired‐end 150‐bp sequencing was performed on an Illumina NovaSeq. Antibodies used are listed in Table S9.

### 4.21 ChIP Sequencing and ChIP‐qPCR

ChIP‐seq library preparation was performed as previously described [6, 7]. ChIP assays were performed in hESC‐ECs or mouse tissues using the SimpleChIP Enzymatic Chromatin IP Kit (Cell Signaling Technology, Cat. No. S9003) according to the manufacturer's instructions. Mouse muscle tissues were minced and enzymatically dissociated into single cells. The resulting muscle cells or hESC‐ECs were cross‐linked with 1% formaldehyde (Sigma) for 10 min at room temperature, and the reaction was quenched with 0.125 M glycine for 5 min. Crosslinked chromatin was fragmented with 0.5 µL Micrococcal Nuclease (CST) for 20 min at 37°C, followed by sonication (Shanghai Lichen Bangxi Technology Co., Ltd.) for three cycles (20s each, peak power 250 W, power ratio 20%). Ten percent of the sheared chromatin was saved as input DNA. The remainder was incubated overnight at 4°C with rotation with the respective antibody. Immune complexes were captured with ChIP‐Grade Protein G Magnetic Beads (CST, Cat. No. 9006). Beads were washed three times with low‐salt buffer (5 min each) and once with high‐salt buffer for 5 min. ChIP Elution Buffer (CST, Cat. No. 7009) was added to input and ChIP samples and incubated at 65°C for 30 min with gentle vortexing, followed by addition of 6 µL 5 M NaCl and 2 µL Proteinase K (CST, Cat. No. 10012) and incubation at 65°C for 2 h. For ChIP‐seq, immunoprecipitated DNA was resuspended in 20 µL nuclease‐free water and libraries were prepared with the NEBNext Ultra DNA Library Prep Kit for Illumina (NEB, Cat. No. E7645). Sequencing was performed as 75 bp paired‐end reads on an Illumina HiSeq 4000. For ChIP‐qPCR, precipitated DNA was resuspended in 50 µL DNA Elution Buffer and diluted to 120 µL. ChIP DNA was analyzed by qPCR using specific primers, and enrichment was normalized to input DNA. Results represent three independent experiments. Antibodies used are listed in Table S9; and primers used in this study are listed in Table S10.

### 4.22 Sequencing Analysis

#### 4.22.1 Single‐Nucleus RNA‐seq Analysis

Reference snRNA‐seq data were analyzed and visualized in R using Seurat (v4.1.1). Standard preprocessing, Uniform Manifold Approximation and Projection (UMAP) dimensional reduction, graph‐based clustering, identification of overexpressed genes and hierarchical clustering were performed following the Seurat workflow. Gene expression distributions were visualized using VlnPlot().

#### 4.22.2 Bulk RNA‐seq Analysis

Sequenced reads were aligned to the mouse reference genome (GRCm39) with the GENCODE mouse annotation M29 (www.gencodegenes.org/) and Spliced Transcripts Alignment to a Reference (STAR; v2.7.10a; https://github.com/alexdobin/STAR) with default parameters. Gene‐level expression was quantified with RSEM (v1.3.1; deweylab.github.io/RSEM/) across all samples. Differential expression was performed using edgeR (v3.38.1; bioconductor.org/packages/release/bioc/html/edgeR.html) using thresholds of absolute log2 fold change ≥ 0.5849 (1.5‐fold) and FDR <0.05. Differentially expressed genes from ECs of heterozygous knockout versus control mice were functionally annotated for Gene Ontology (GO) and Kyoto Encyclopedia of Genes and Genomes (KEGG) pathways using DAVID Bioinformatics Tool (v2021; david.ncifcrf.gov/).

#### 4.22.3 Murine ChIP‐seq / CUT&Tag‐seq / CUT&RUN‐seq Analysis

Reads were aligned to GRCm39 using Burrow‐Wheeler Aligner (v0.7.17) with the Maximum Exact Match algorithm, and downstream annotations referenced GENCODE M29. After removal of GRCm39 blacklist regions and duplicate reads, peaks were called with Model‐Based Analysis of ChIP‐Seq peak caller (MACS; v2.2.8; pypi.org/project/MACS2/) at a q‐value cutoff of 0.01. Peaks were annotated with Hypergeometric Optimization of Motif Enrichment (HOMER, v4.11.1; homer.ucsd.edu/homer/) using GENCODE M29, assigned to the nearest transcription start site (default), and classified into TSS (−1 kb to +100 bp), transcription termination site (TTS, −100 bp to +1 kb), exonic, intronic, and intergenic categories. Peaks associated with protein‐coding genes were retained for integrative analyses, and selected loci were visualized in the UCSC Genome Browser.

#### 4.22.4 Histone ChIP‐seq Analysis

Processed peak files for H3K4me3, H3K27me3 and H3K27ac in HUVECs were retrieved from the Encyclopedia of DNA Elements (ENCODE project; https://www.encodeproject.org/), and peaks for selected genes were visualized using the UCSC Genome Browser.

### 4.23 Statistical Analysis

Data are presented as mean ± SEM, and all analyses were conducted in a blinded manner. Differences between two groups were assessed using unpaired two‐tailed *t*‐tests. For comparisons involving three or more groups with normal distributions and equal variances, one‐way ANOVA followed by *Tukey's* multiple comparisons test was used. For experiments with two independent factors, two‐way ANOVA with *Tukey's* multiple comparisons test was applied unless otherwise specified. Group sizes and specific statistical tests are detailed in the figure legends. Statistical significance was defined as *p* <0.05. All analyses were conducted using GraphPad Prism.

### 4.24 Resource Availability

All sequencing data generated in this study are publicly available. Bulk RNA‐seq from *Cdh5‐Cre;Yy1^fl/+^* and *Yy1^fl/fl^* mice is deposited at NCBI Gene Expression Omnibus under GSE331193, and YY1 ChIP‐seq/CUT&RUN‐seq from ECs is available under GSE331194 and GSE331195. Bulk RNA‐Seq of CD45^−^CD31^+^ ECs from ischemic muscles of *Lepr^db/db^* and *Lepr^db/+^* mice is available in Mendeley Data (DOI: 10.17632/4n2whfwvd6.1). Reference HUVEC histone ChIP‐seq datasets are from ENCODE: ENCSR000AKN (H3K4me3), ENCSR000AKK (H3K27me3), and ENCSR000ALB (H3K27ac). Reference murine EC datasets are from Gene Expression Omnibus: GSM8496872 (H3K27ac CUT&Tag‐seq), GSM4904892 (H3K27me3 ChIP‐seq). The reference human gastrocnemius snRNA‐seq dataset is GSE233882. Analysis code is available upon reasonable request.

## Author Contributions

H.Q., J.T., L.C., Y.H., C.L., and W.Y. performed experiments and analysed the data. C.K.H. analysed the bioinformatics data. R.H.L.W. provided critical suggestions. S.H. and B.Z. provided mouse lines. K.O.L. designed the research, interpreted the data, and wrote the manuscript.

## Conflicts of Interest

The authors declare no conflicts of interest.

## Supporting information




**Supporting file**: advs75735‐sup‐0001‐SuppMat.pdf

## Data Availability

The data that support the findings of this study are available on request from the corresponding author. The data are not publicly available due to privacy or ethical restrictions.
