# Supplementary material for: CD4+ Tregs Drive Post‐Ischemic Sprouting Angiogenesis via Endothelial YY1/MAML1 Reactivation
Source: Adv Sci (Weinh). 2026 May 20;13(45):e18564. doi: 10.1002/advs.202518564 (PMC13336064; doi:10.1002/advs.202518564)
Supplement: Supplementary file 1 [file ADVS-13-e18564-s001.pdf]

Fig. S1

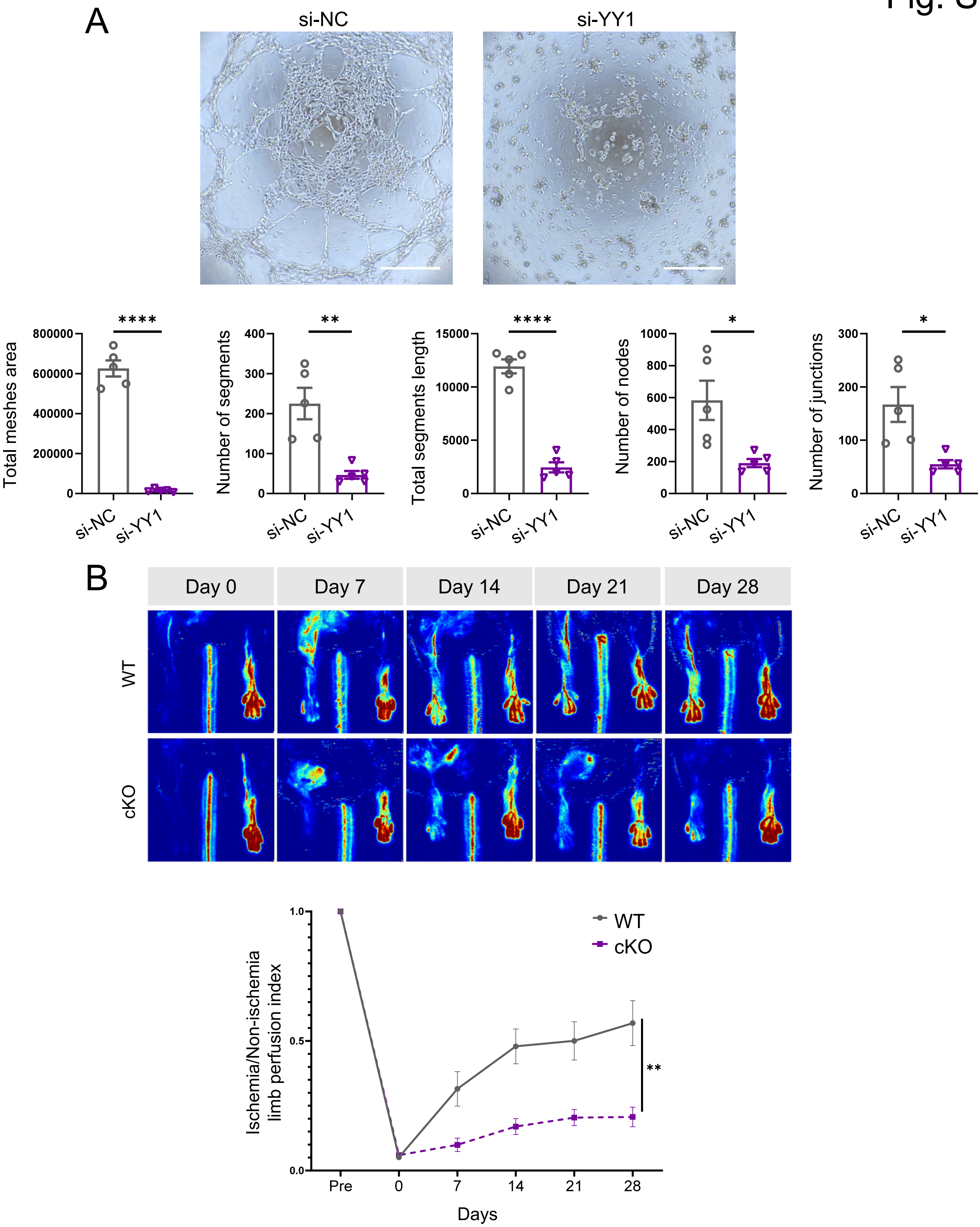

Figure S1. Endothelial YY1 is required for angiogenesis both *in vitro* and *in vivo*

(A) Representative microscopy images and quantification of tube formation assays in si-NC and si-YY1 treated hESC-ECs. Cells were cultured on Matrigel for 6 hours before imaging (n = 5/group). *P < 0.05, **P < 0.01, ****P < 0.0001. (n = 5). Scale bars: 1mm.

(B) Representative laser Doppler images and quantification of hindlimb blood perfusion comparing WT (*Cdh5-CreER*) and cKO (*Cdh5-CreER;Y1*$^{fl/fl}$). **P < 0.01. (n = 6).

Data are presented as mean ± SEM. Statistical significance was determined by unpaired *t*-test (A, B).

Fig. S2

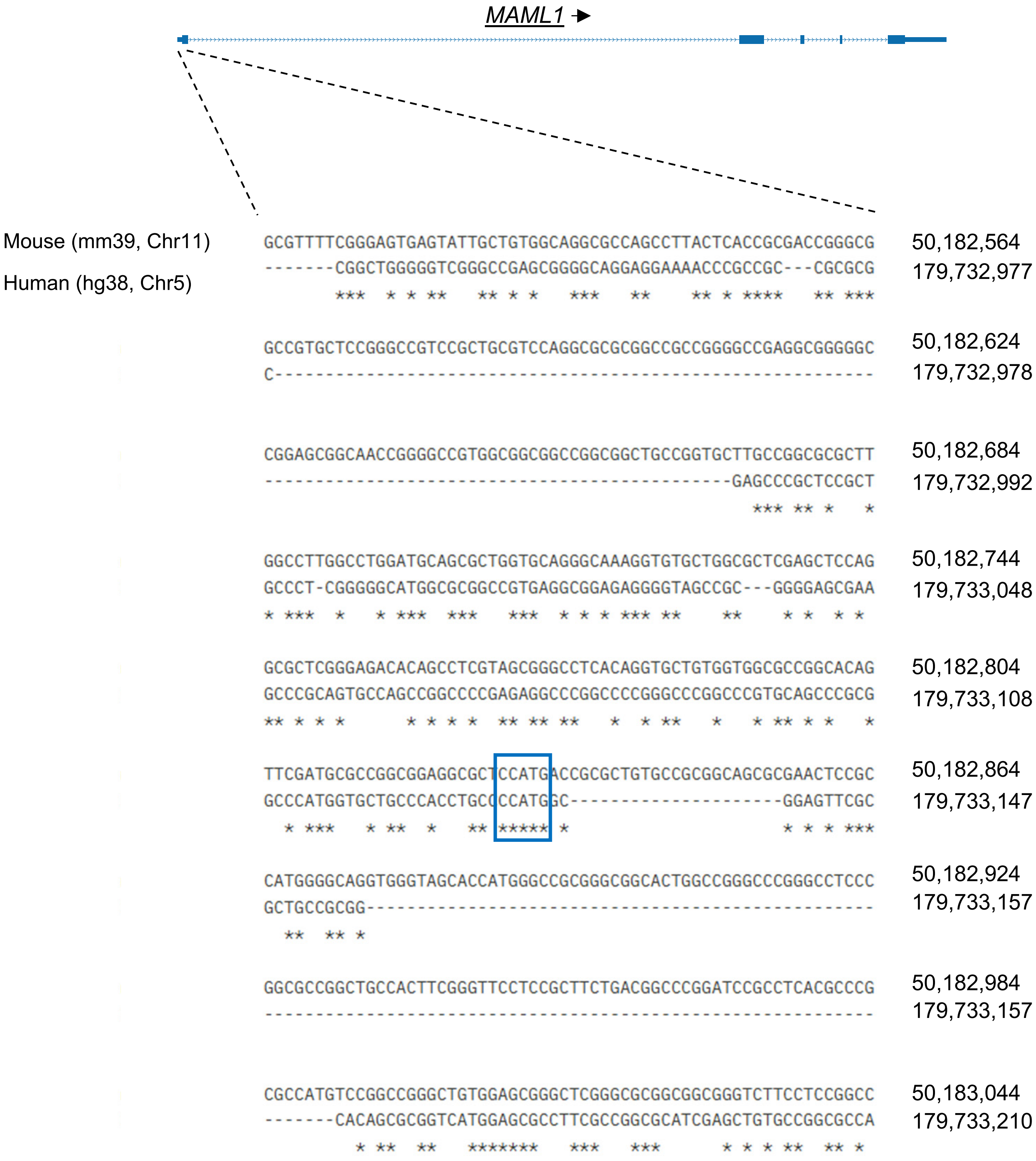

Figure S2. The canonical binding site of YY1 in the putative promoter regions of *Maml1* are conserved across human and mouse. A portion of alignment of orthologous regions between *Maml1* putative promoter regions in human (hg38) and mouse (mm39) genomes was determined by Clustal Omega. Asterisks (*) denote identical nucleotides between the two DNA sequences. Blue box indicates the core motif sequence of YY1.

Fig. S3

A

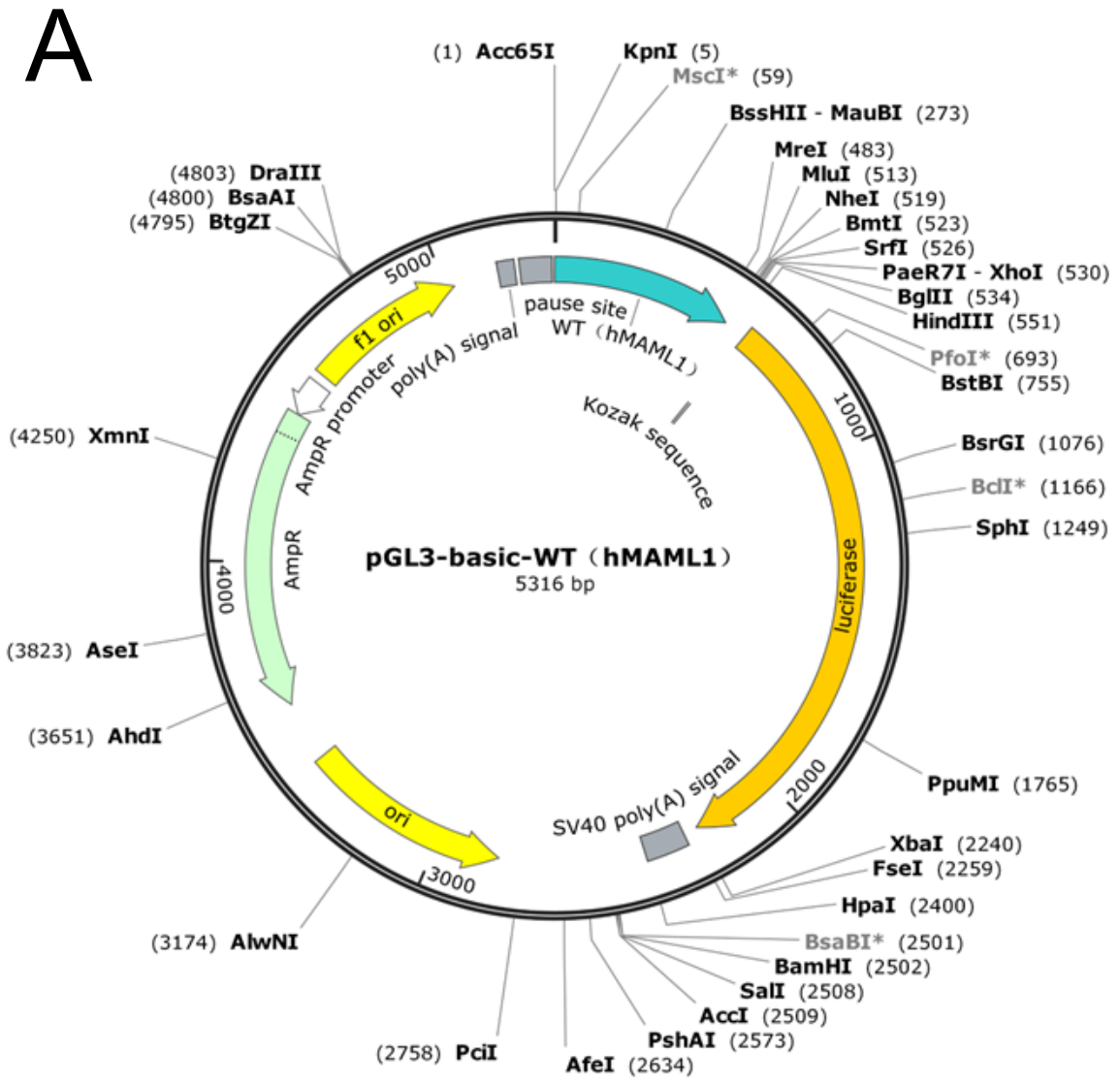

Predicted YY1 binding region in MAML1 promoter
hg38 chr5:179,732,707 - 179,733,212

TTGCGTGCTCGTCCCCACGCCCTCGGGCGGCCGGTTCTTCCTTCTCTGATT
GGCCAGGGCCCCTGCTCATCAGTCGTGATGCACAAGGTCTCTTACCGAGGC
site 1 CCCGCCTTCTTCCGAGAGGCCCGAAAACAATTTTAAGATGGCGGCCGCGGC
site 2 GGTAGCGCGGAAAACAATGGGGCCGGGGCGGTGGGAGAGGCCGAGGCT
TGAGGTAGGCAGCAAGCGCCGGCTGGGGGTCGGGCCGAGCGGGGCAGGA
GGAAAACCCGCCGCCGCGCGCGAGCCCGCTCCGCTGCCCTCGGGGGCAT
GGCGCGGCCGTGAGGCGGAGAGGGGTAGCCGCGGGGAGCGAAGCCCGC
AGTGCCAGCCGGCCCCGAGAGGCCCGGCCCCGGGCCCGGCCCGTGCAGC
CCGCGGCCCATGGTGCTGCCCACCTGCCCCATGGCGGAGTTCGCGCTGCC
GCGGCACAGCGCGGTCATGGAGCGCCTTCGCCGGCGCATCGAGCTGTGCC
GGCGCCACC

B

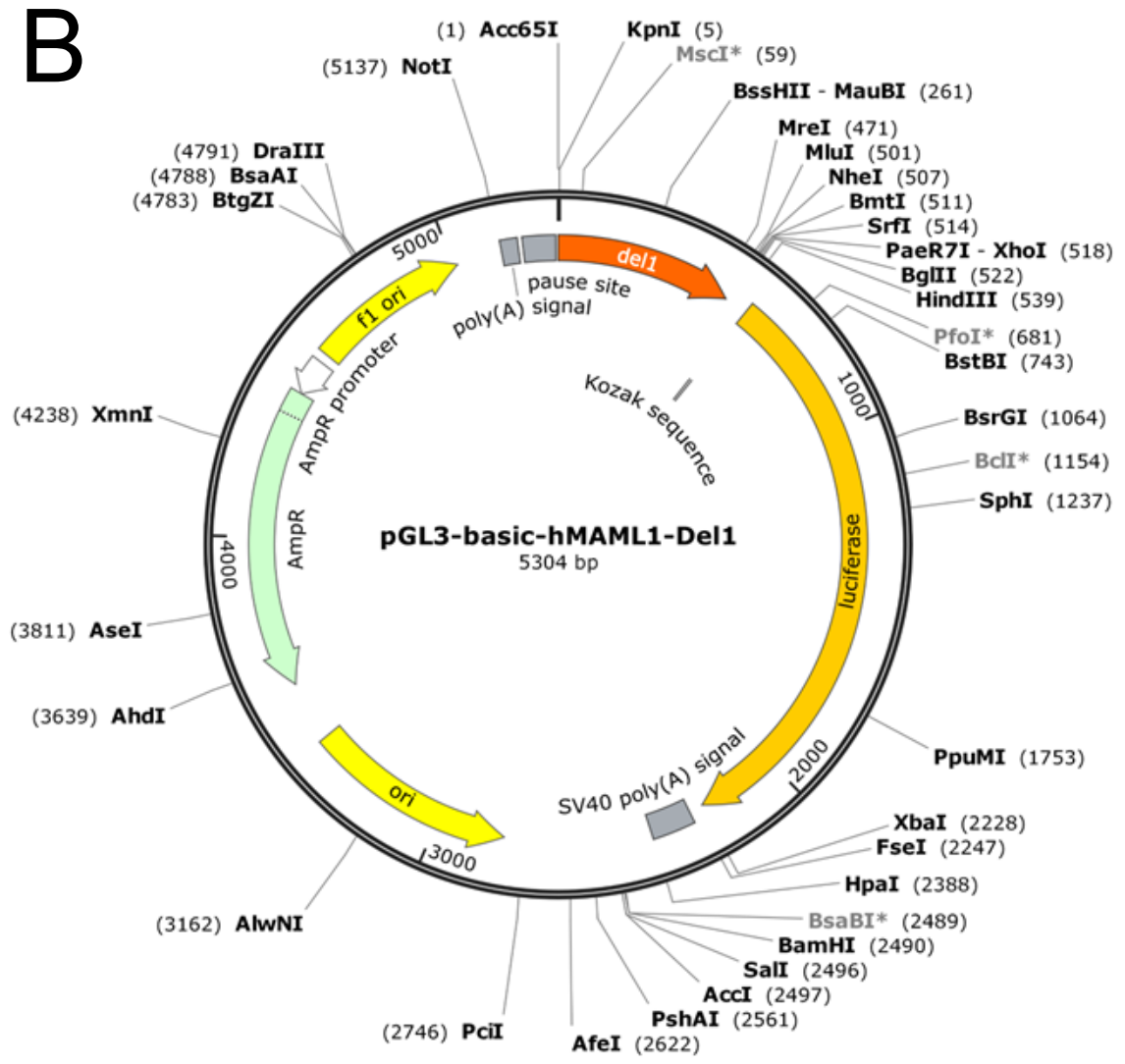

Predicted YY1 binding region in MAML1 promoter
hg38 chr5:179,732,707 - 179,733,212

TTGCGTGCTCGTCCCCACGCCCTCGGGCGGCCGGTTCTTCCTTCTCTGATT
GGCCAGGGCCCCTGCTCATCAGTCGTGATGCACAAGGTCTCTTACCGAGGC
~~site 1~~ CCCGCCTTCTTCCGAGAGGCCCGAAAACAATTT~~TAAGATGGCGGC~~CGCGGC
site 2 GGTAGCGCGGAAAACAATGGGGCCGGGGCGGTGGGAGAGGCCGAGGCT
TGAGGTAGGCAGCAAGCGCCGGCTGGGGGTCGGGCCGAGCGGGGCAGGA
GGAAAACCCGCCGCCGCGCGCGAGCCCGCTCCGCTGCCCTCGGGGGCAT
GGCGCGGCCGTGAGGCGGAGAGGGGTAGCCGCGGGGAGCGAAGCCCGC
AGTGCCAGCCGGCCCCGAGAGGCCCGGCCCCGGGCCCGGCCCGTGCAGC
CCGCGGCCCATGGTGCTGCCCACCTGCCCCATGGCGGAGTTCGCGCTGCC
GCGGCACAGCGCGGTCATGGAGCGCCTTCGCCGGCGCATCGAGCTGTGCC
GGCGCCACC

C

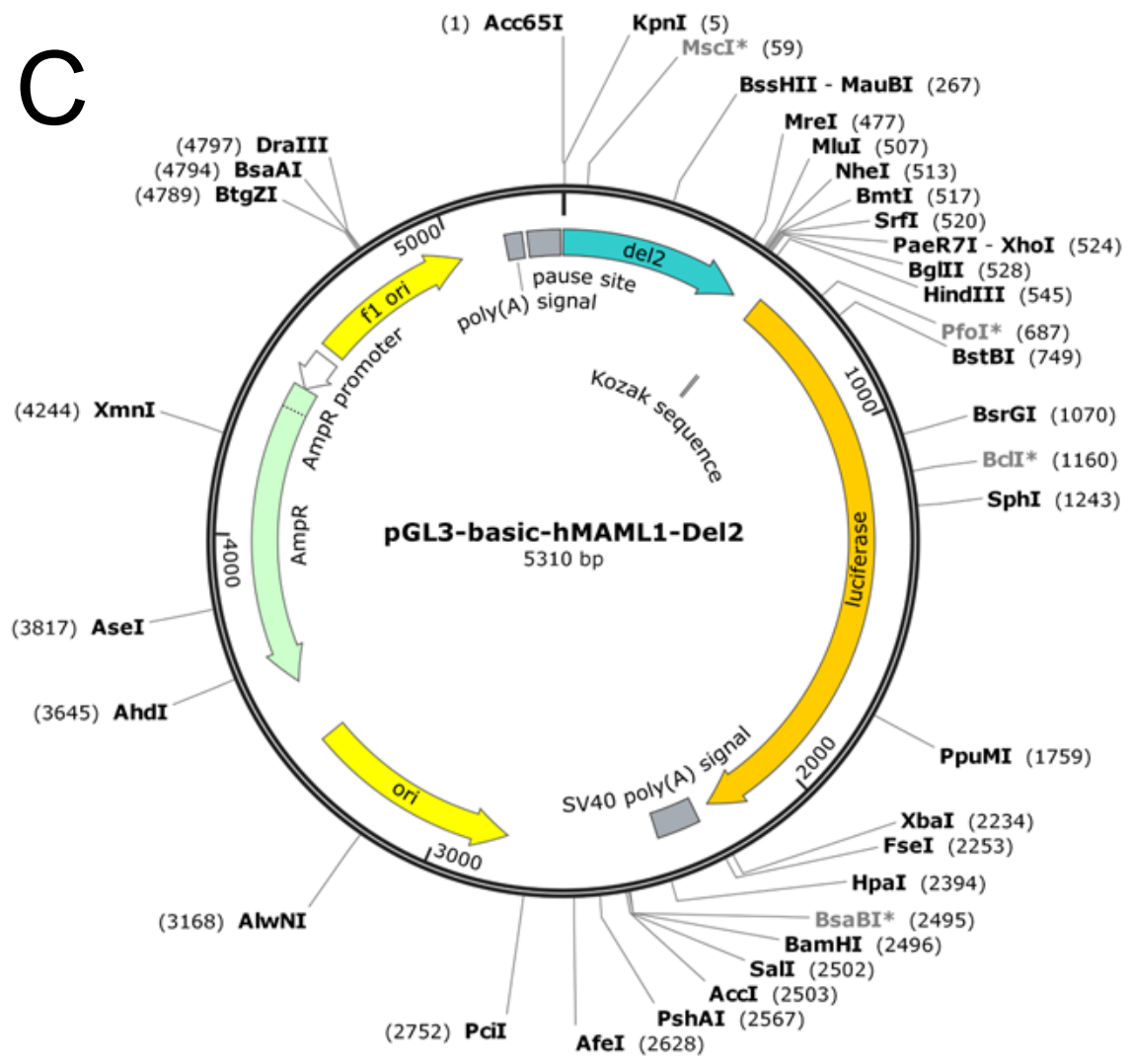

Predicted YY1 binding region in MAML1 promoter
hg38 chr5:179,732,707 - 179,733,212

TTGCGTGCTCGTCCCCACGCCCTCGGGCGGCCGGTTCTTCCTTCTCTGATT
GGCCAGGGCCCCTGCTCATCAGTCGTGATGCACAAGGTCTCTTACCGAGGC
site 1 CCCGCCTTCTTCCGAGAGGCCCGAAAACAATTTTAAGATGGCGGCCGCGGC
~~site 2~~ GGTAGCGCGGAAAAC~~AATGGG~~GCCGGGGCGGTGGGAGAGGCCGAGGCT
TGAGGTAGGCAGCAAGCGCCGGCTGGGGGTCGGGCCGAGCGGGGCAGGA
GGAAAACCCGCCGCCGCGCGCGAGCCCGCTCCGCTGCCCTCGGGGGCAT
GGCGCGGCCGTGAGGCGGAGAGGGGTAGCCGCGGGGAGCGAAGCCCGC
AGTGCCAGCCGGCCCCGAGAGGCCCGGCCCCGGGCCCGGCCCGTGCAGC
CCGCGGCCCATGGTGCTGCCCACCTGCCCCATGGCGGAGTTCGCGCTGCC
GCGGCACAGCGCGGTCATGGAGCGCCTTCGCCGGCGCATCGAGCTGTGCC
GGCGCCACC

Figure S3. Construction of *MAML1* luciferase reporter plasmids.
(A) Schematic of the wildtype (WT) luciferase reporter construct (pGL3-basic-wtMAML1), containing the putative YY1 binding region (indicated) cloned upstream of the luciferase gene. The corresponding nucleotide sequence is shown on the right, with key promoter elements color-coded. (B, C) Schematics and sequences of two deletion reporters (Del1/ΔSite1 and Del2/ΔSite2). In each mutant, a segment within the predicted YY1 binding region (highlighted in red in the wildtype sequence) was deleted to assess its role in YY1-mediated transcriptional regulation.

Fig. S4

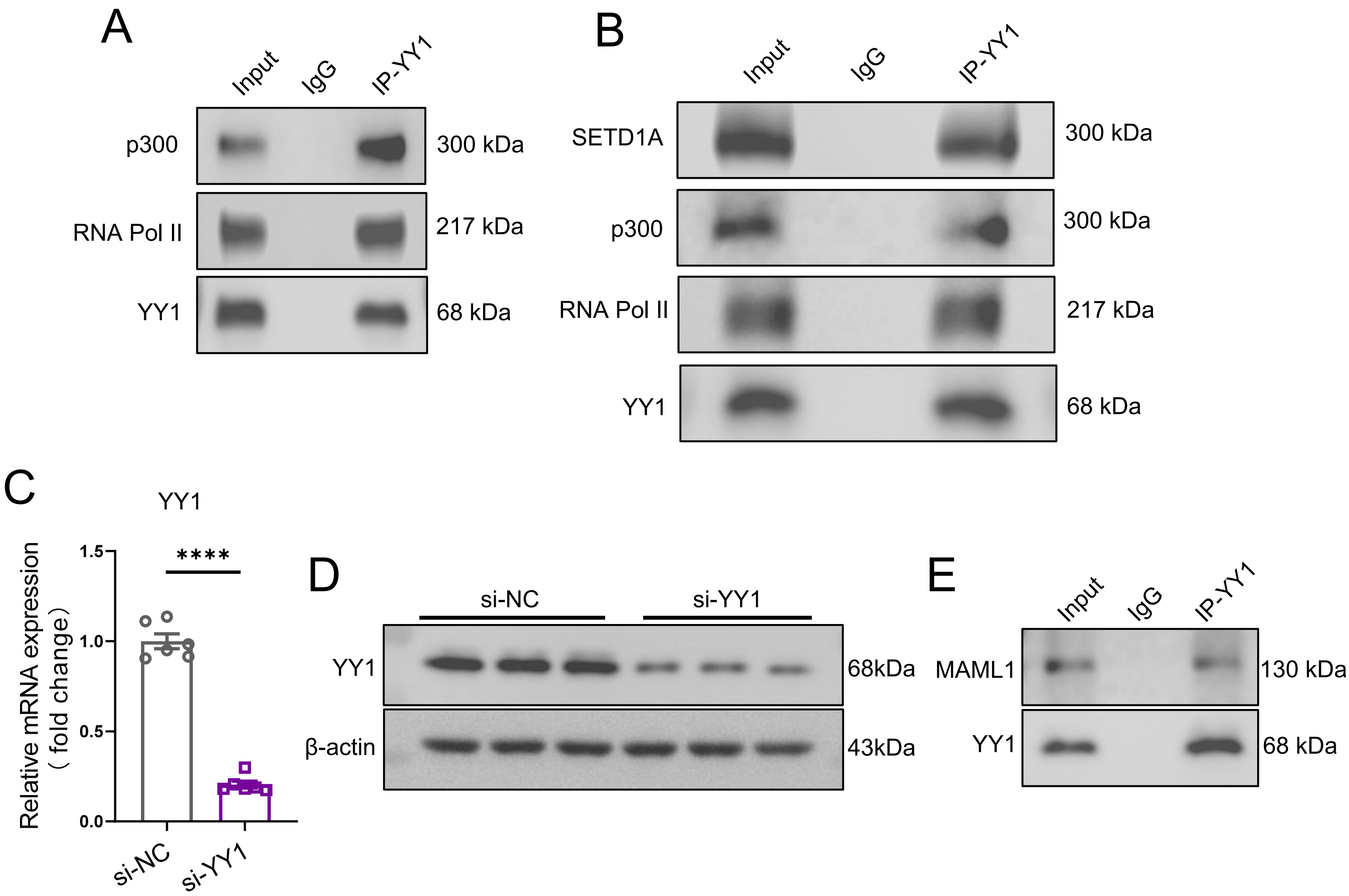

Figure S4. YY1 interacts with epigenetic regulators and transcriptional machinery in endothelial cells

(A) Co-IP of YY1 with SETD1A, p300, RNA Pol II in hESC-ECs using an anti-YY1 antibody.

(B) Co-IP of YY1 with SETD1A, p300, RNA Pol II in isolated lung ECs using an anti-YY1 antibody.

(C) Relative mRNA expression level of YY1 in hESC-ECs transfected with si-NC or si-YY1 for 48h. ****P < 0.0001. (n = 6).

(D) Western blot analysis of YY1 protein expression in hESC-ECs treated as in (C).

(E) Co-IP of YY1 with MAML1 in hESC-ECs using an anti-YY1 antibody.

Data are presented as mean ± SEM. Statistical significance was determined by unpaired *t*-test (C).

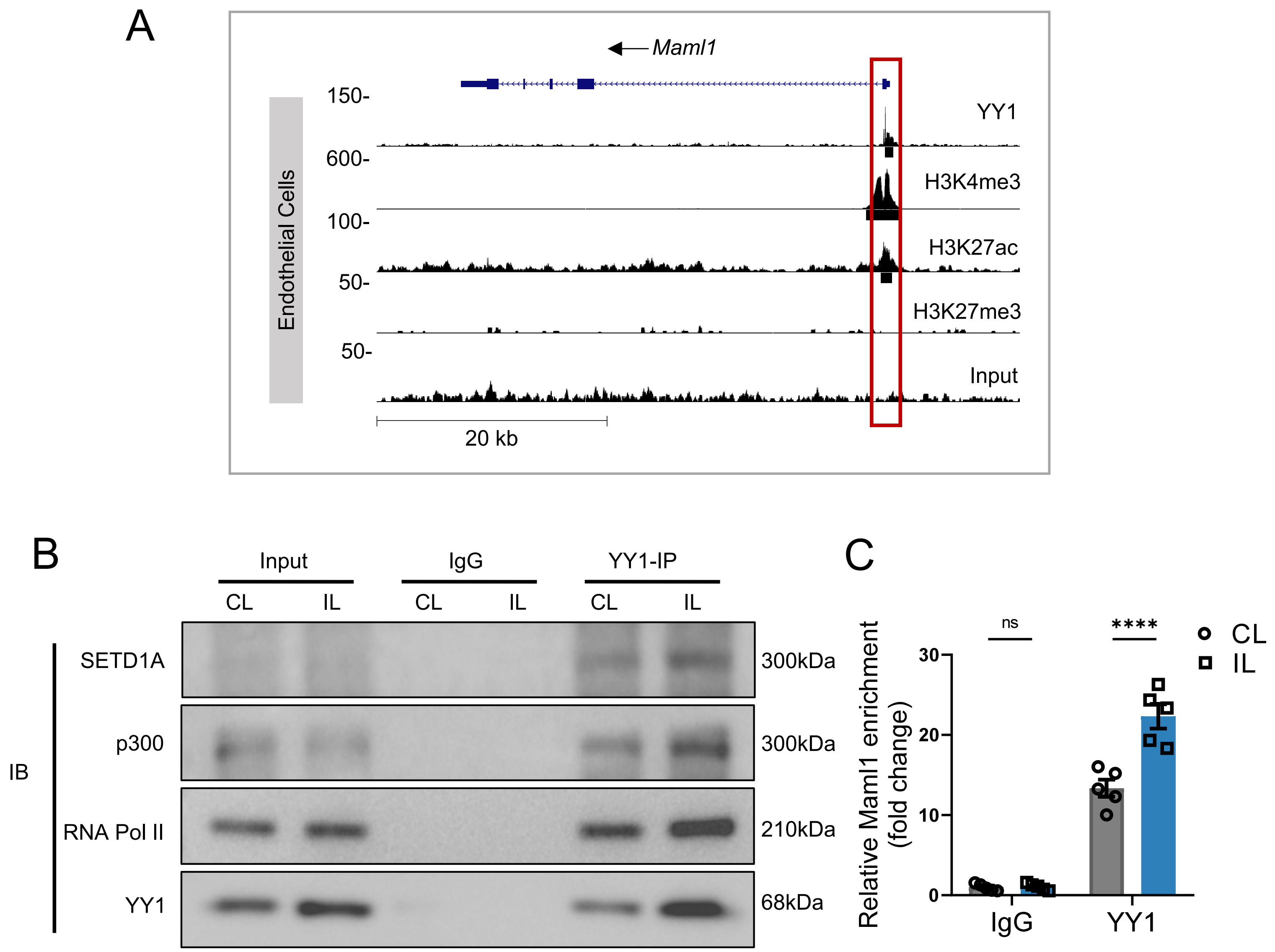

Figure S5. Ischemia increases YY1 interaction with SETD1A-p300-RNA pol II and YY1 occupancy

(A) Genome snapshot of YY1, H3K4me3, H3K27ac and H3K27me3 ChIP-seq, CUT&Tag-seq or CUT&RUN-seq using mouse endothelial cells at Maml1 gene locus.

(B) Co-IP of SETD1A, p300, RNA polymerase II (RNA Pol II), and YY1 proteins in mouse hind limb endothelial cells under control limb (CL) and ischemic limb (IL) conditions.

(C) ChIP-quantitative PCR analysis for YY1 binding to the putative MAML1 promoter. Chromatin was extracted from mouse hind limb endothelial cells from CL and IL and then precipitated with an anti-YY1 antibody or IgG (negative control). ****$P < 0.0001$. ($n = 5$).

Data are presented as mean ± SEM. Statistical significance was determined by two-way ANOVA with *Tukey*'s multiple comparisons test (C).

Fig. S6

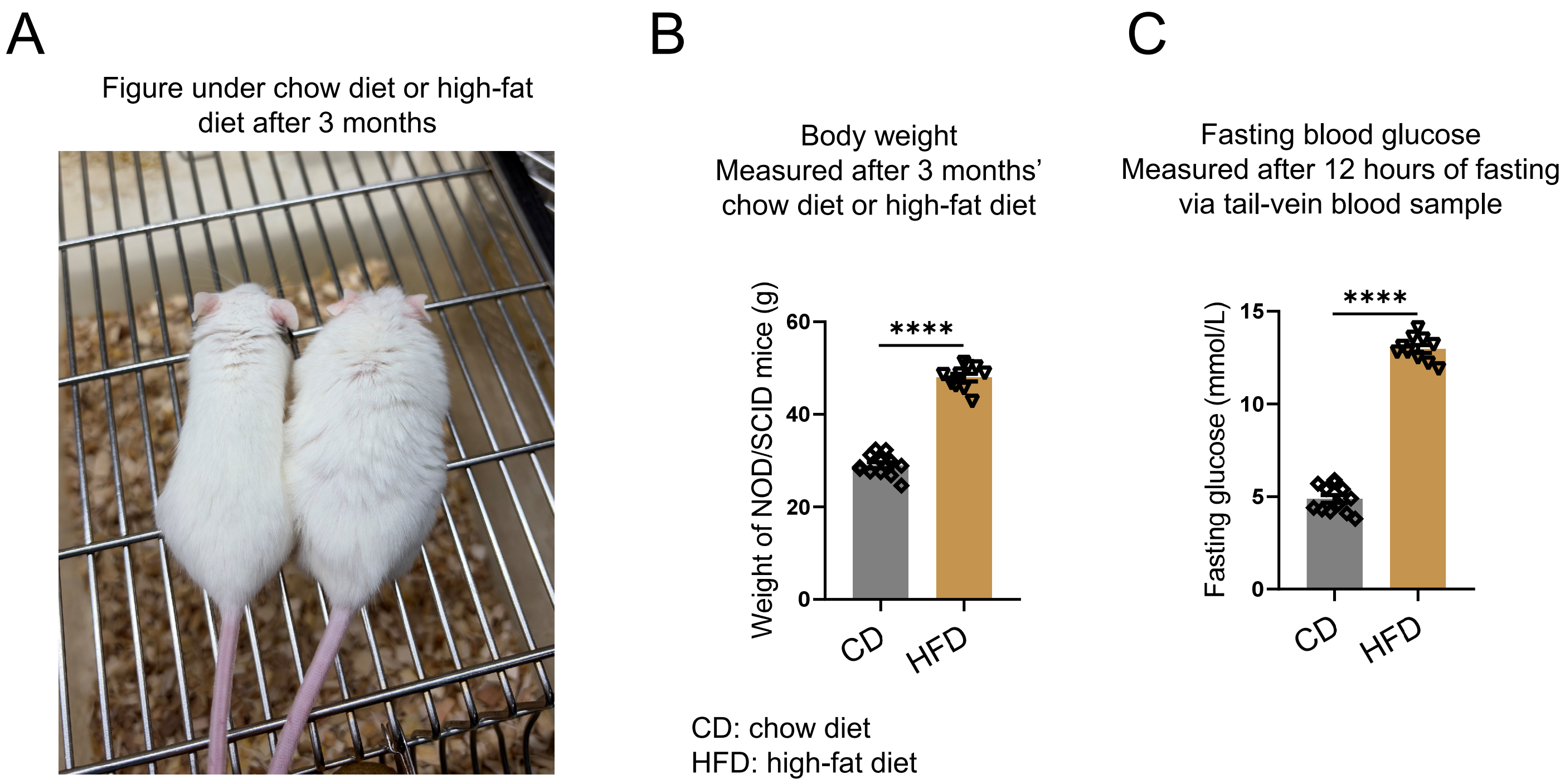

Figure S6. Establishment of T2D in NOD.SCID mice

(A) Representative figure of NOD/SCID mouse under chow diet or high-fat diet after 3 months.

(B) Quantification of NOD/SCID mice body weight measured after 3 months' chow diet or high-fat diet. ****$P < 0.0001$. ($n = 10$-12).

(C) Quantification of NOD/SCID mice fasting blood glucose measured after 12 hours of fasting via tail-vein blood sample after 3 months' chow diet or high-fat diet. ****$P < 0.0001$. ($n = 10$-12).

Data are presented as mean ± SEM. Statistical significance was determined by unpaired *t*-test (B, C).

Fig. S7

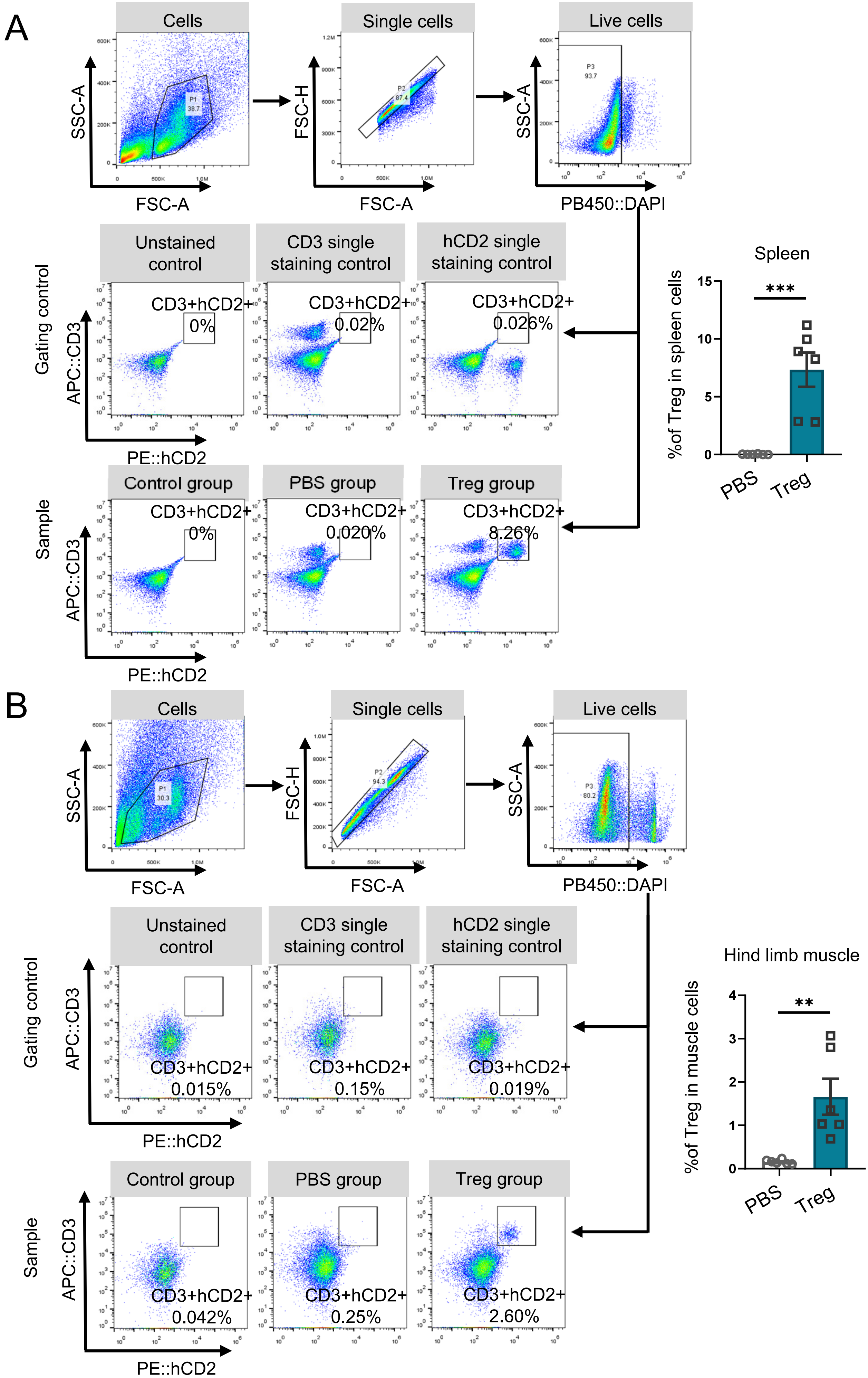

Figure S7. Adoptive transfer of $CD3^{+}hCD2^{+}$ cells increases their infiltration into the spleen and hindlimb muscle. (A, B) Flow cytometry gating strategy for $CD3^{+}hCD2^{+}$ cells in the spleen (A) and hindlimb muscle (B). Representative plots show unstained, single-stained (CD3 or hCD2), and double-stained ($CD3^{+}hCD2^{+}$) populations. Quantification of $CD3^{+}hCD2^{+}$ cells in spleen or hind limb muscle. $^{**}P < 0.01$, $^{***}P < 0.001$. ($n = 6$). Data are mean ± SEM. Statistical analysis was performed using unpaired *t*-test (A, B).

Fig. S8

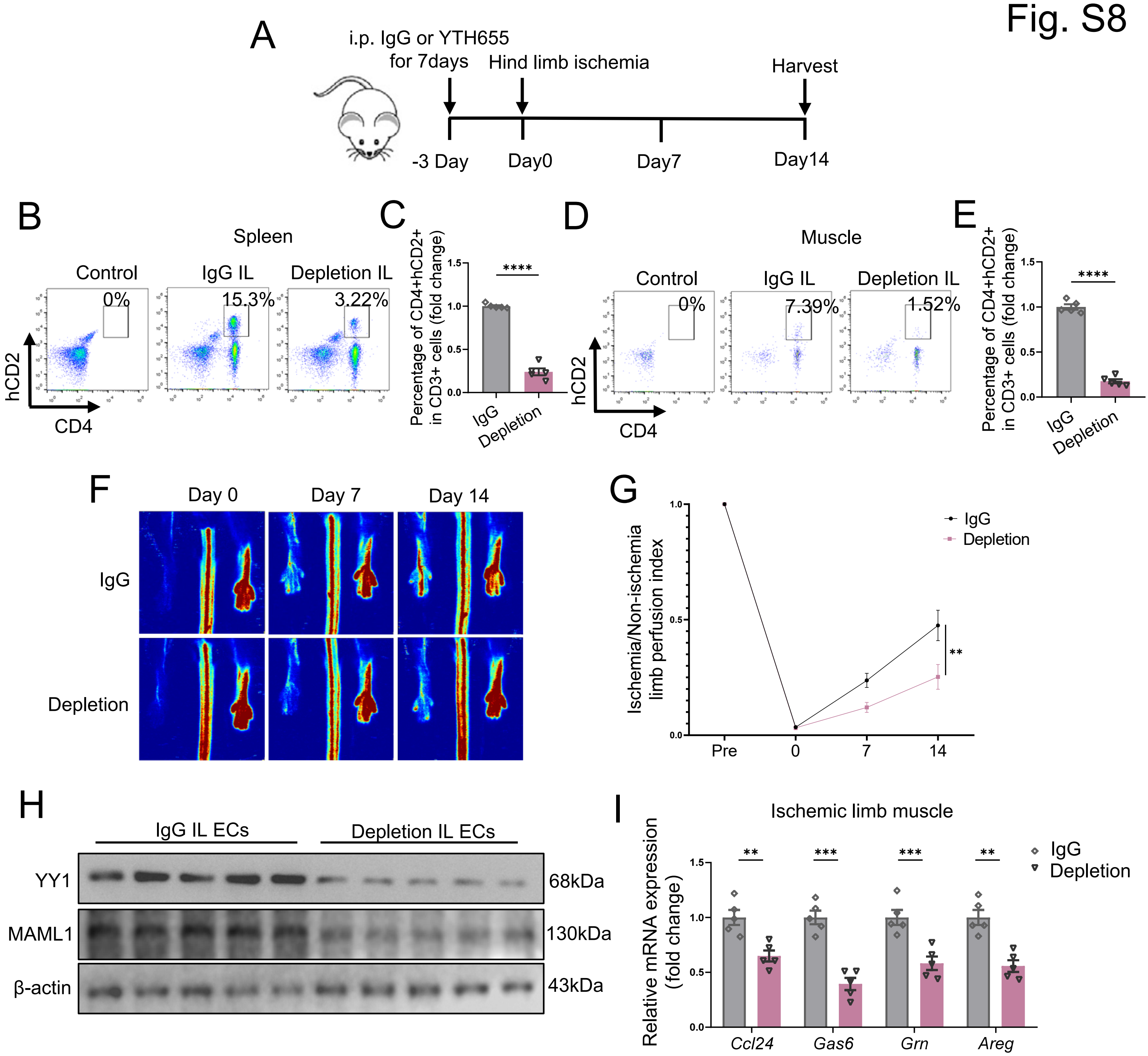

Figure S8. CD4⁺ Treg depletion impairs post-ischemic reperfusion

(A) Schematic of the experimental timeline for in vivo Treg depletion and hindlimb ischemia model. Mice were treated with anti-human CD2 antibody (clone YTH655) 250ug or IgG control via intraperitoneal injection for 7 days, and 3 days prior to HLI surgery, and tissues were harvested for analysis at day 14 post-ischemia. (B) Representative flow cytometry plots showing the gating strategy for identifying $CD4^+hCD2^+$ amount $CD3^+$ in the spleen under control (IgG) and YTH655 (Depletion) conditions. (C) Representative flow cytometry plots showing the frequency of $CD4^+hCD2^+$ cells amount $CD3^+$ in the spleen. (D, E) Representative flow cytometry plots and quantification of $CD4^+hCD2^+$ cells amount $CD3^+$ in the hind limb muscle. ****P < 0.0001. (n = 5). (F) Representative laser Doppler perfusion images of hindlimbs at the indicated time points (pre-ischemia and day 0, 7, 14 post-ischemia). (G) Representative laser Doppler images and quantification of hindlimb blood perfusion rate. **P < 0.01. (n = 5). (H) Western blot analysis of MAML1 and YY1 protein expression in endothelial cells (ECs) isolated from the ischemic limb muscle of IgG and Depletion mice. β-actin serves as a loading control. (I) Quantitative real-time PCR analysis of *Ccl24*, *Gas6*, *Grn* and *Areg* mRNA expression in ischemic limb muscle tissues from IgG and Depletion mice. **P < 0.01, ***P < 0.001. (n = 5). Data are presented as mean ± SEM.. Statistical significance was determined by unpaired *t*-test (C, E, I) or two-way ANOVA with *Tukey's* post-hoc test (G).

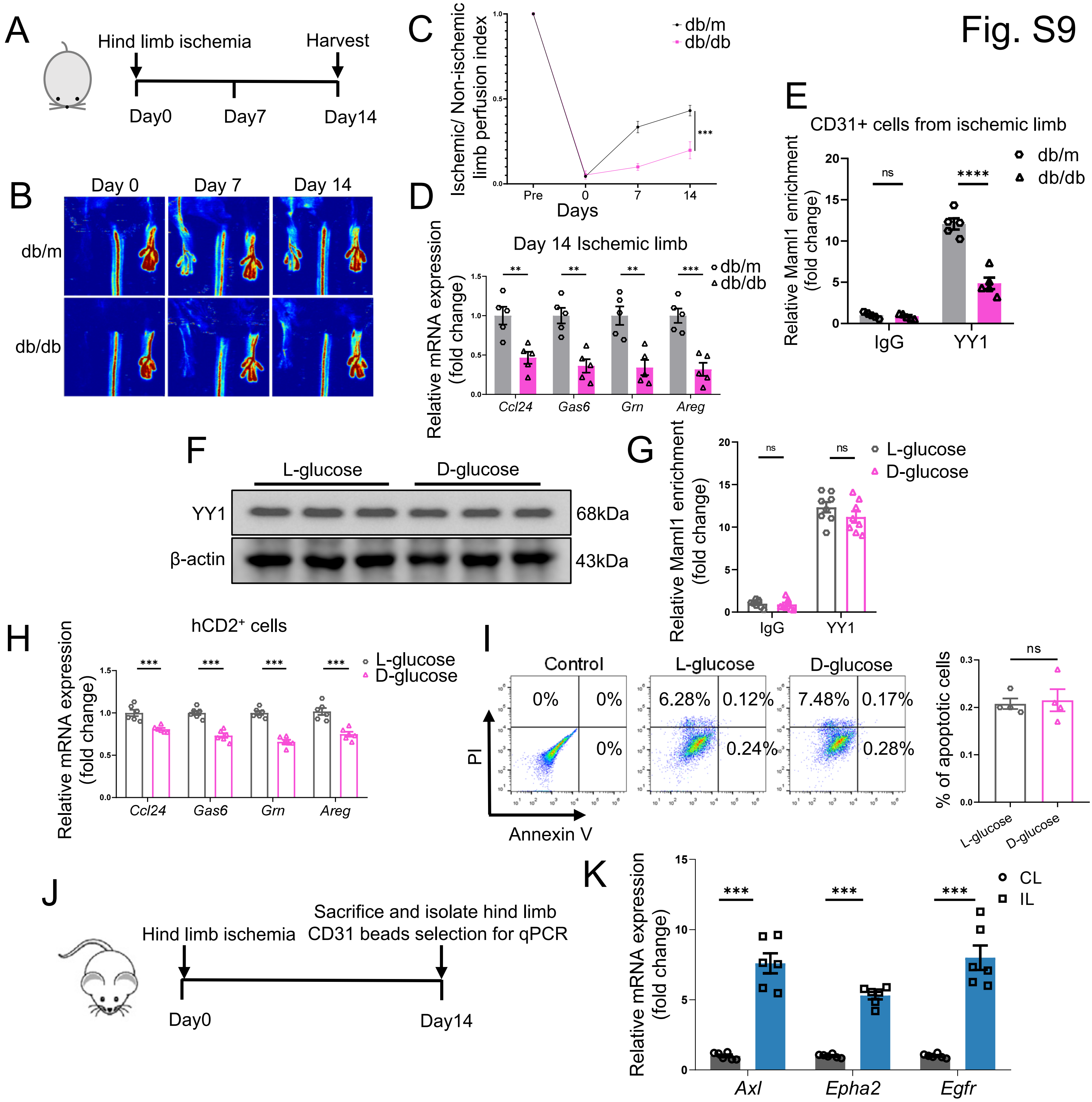

Figure S9. Ischemia induces vascular regeneration and Treg effects that are blunted in diabetes. (A) Schematics of experimental design. (B) Representative laser Doppler perfusion images. (C) Quantification of ischemic/non-ischemic limb perfusion rate. ***P < 0.001. (n = 5). (D) Quantitative real-time PCR analysis of *Ccl24*, *Gas6*, *Grn* and *Areg* mRNA expression in ischemic limb muscle tissues from db/m and db/db mice. **P < 0.01, ***P < 0.001. (n = 5). (E) ChIP-quantitative PCR analysis for YY1 binding to the putative MAML1 promoter. Chromatin was extracted from mouse hind limb endothelial cells from db/m and db/db mice and then precipitated with an anti-YY1 antibody or IgG (negative control). ****P < 0.0001. (n = 5). (F) Western blot analysis of YY1 protein expression in hESC-ECs treated with 25 mM L-glucose or D-glucose for 72 hours. β-actin serves as a loading control.(G) ChIP-quantitative PCR analysis for YY1 binding to the putative MAML1 promoter. Chromatin was extracted from hESC-ECs under (F) condition and then precipitated with an anti-YY1 antibody or IgG. ns, no significant difference. (H) Activated hCD2⁺ Tregs purified from NOD.Foxp3hCD2 mice were cultured in 25 mM L- or D-glucose for 72 hours then for qPCR analysis. ***P < 0.001. (n = 6). (I) Flow cytometry were harvested from hCD2+ cells under (H) condition, ns, no significant difference. (J) Schematic of the experimental procedure for isolating endothelial cells from ischemic hindlimbs at day 14 for qPCR analysis. (K) Quantitative real-time PCR analysis of *Axl*, *Epha2*, *Egfr* mRNA expression in hind limb muscle ECs from CL or IL. ***P < 0.001. (n = 5). Data are mean ± SEM.. Statistical significance was determined by two-way ANOVA with *Tukey's* post-hoc test (C) or unpaired t-test (D, E, G, H, I, K).

Fig. S10

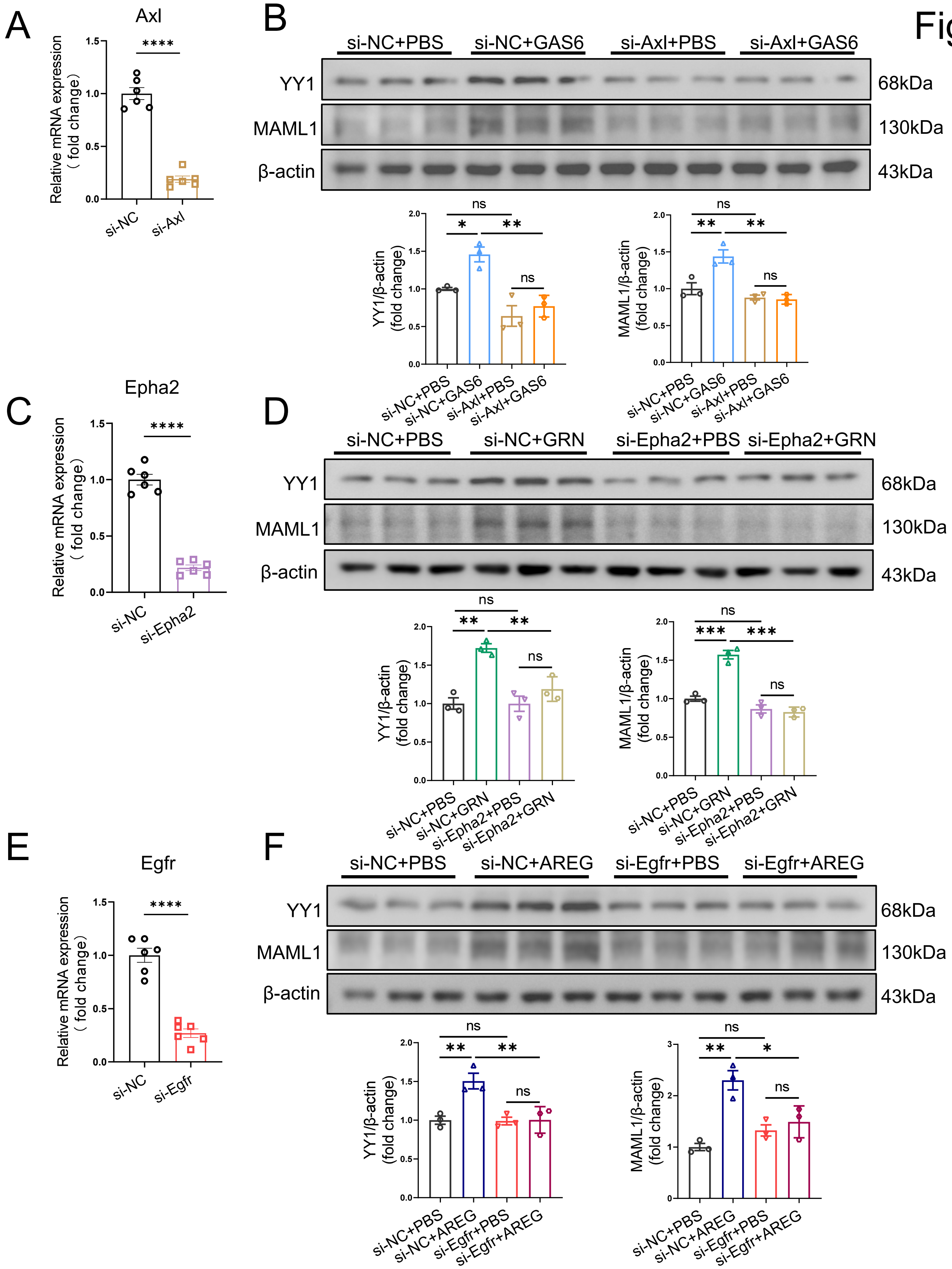

Figure S10. GAS6, GRN and AREG induce endothelial YY1/MAML1 expression through their respective receptors in ECs. (A) Axl mRNA levels in mouse lung ECs transfected with si-NC or si-Axl. ****P < 0.0001. (n = 6). (B) Western blot and quantification of YY1 and MAML1 levels under transfected with si-NC or si-Axl then stimulated with GAS6 (100 ng/mL) or vehicle for 48 hours. *P < 0.05, **P < 0.01, (ns, no significant difference). (n = 3). (C) Epha2 mRNA levels in mouse lung ECs transfected with si-NC or si-Epha2. ****P < 0.0001. (n = 6). (D) Western blot and quantification of YY1 and MAML1 levels under transfected with si-NC or si-Epha2 then stimulated with GRN (1 ug/mL) or vehicle for 48 hours. **P < 0.01, ***P < 0.001, (ns, no significant difference). (n = 3). (E) Egfr mRNA levels in mouse lung ECs transfected with si-NC or si-Egfr. ****P < 0.0001. (n = 6). (F) Western blot and quantification of YY1 and MAML1 levels under transfected with si-NC or si-Egfr then stimulated with AREG (100 ng/mL) or vehicle for 48 hours. *P < 0.05, **P < 0.01, (ns, no significant difference). (n = 3). Data are presented as mean ± S.E.M.. Each dot represents 1 biological replicate. The p values were calculated by unpaired *t*-test (A, C, E) or two-way ANOVA with *Tukey's* multiple comparisons test (B, D, F).

Fig. S11

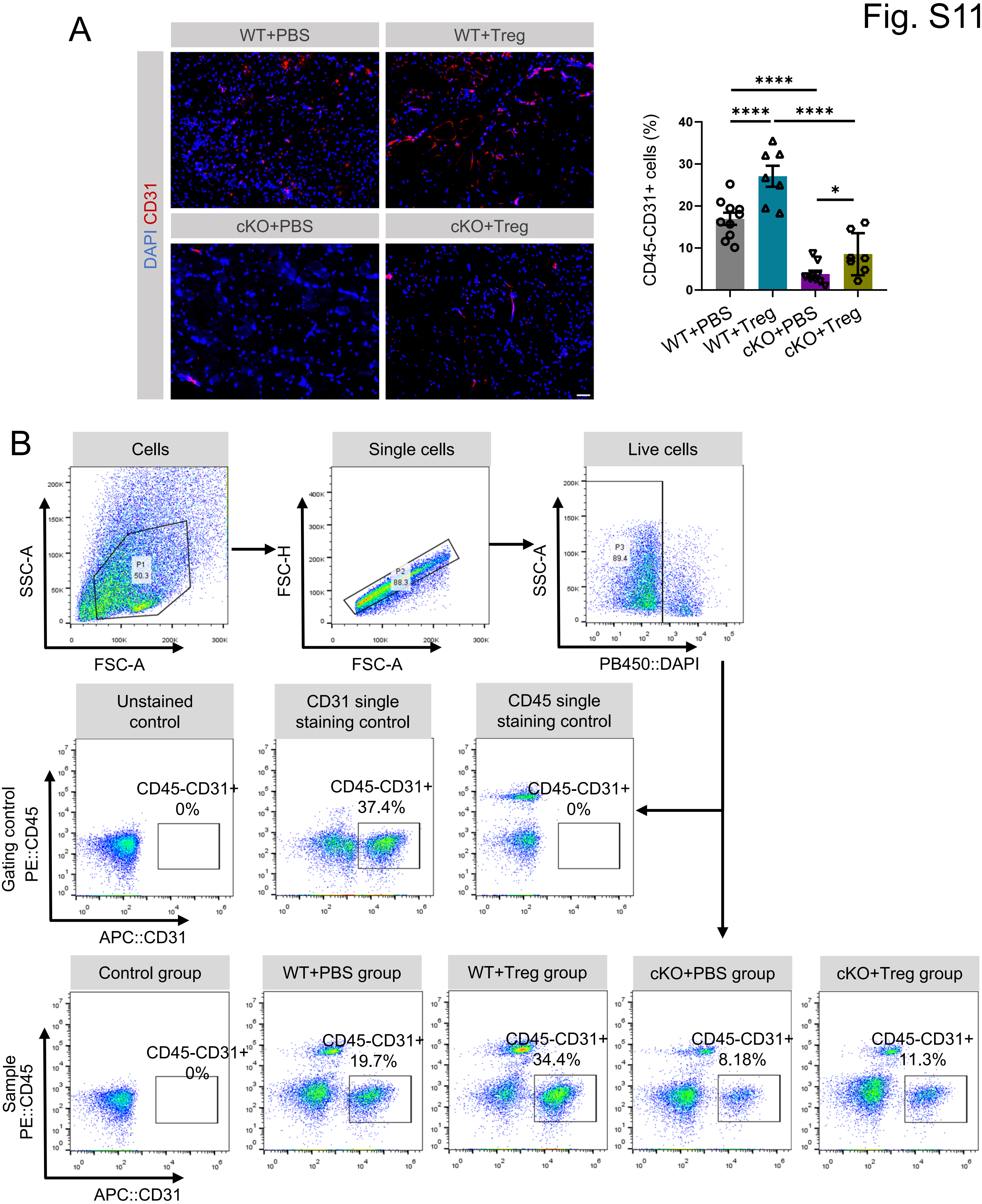

Figure S11. CD4⁺ Tregs promote angiogenesis through endothelial YY1.
(A) Immunohistochemical staining of CD31 in ischemic muscle of WT+PBS, WT+Treg, cKO+PBS, and cKO+Treg mice with quantification (n= 7-10). Scale bar = 40 μm. *P < 0.05, ****P < 0.0001. (B) Flow cytometry gating strategy for $CD45^{-}CD31^{+}$ endothelial cells from ischemic muscle. Representative plots show percentage of different groups described in Figure 7F. WT: *Cdh5-CreER*; cKO: *Cdh5-CreER;Yyl-fl/fl*
Data are presented as mean ± S.E.M.. Each dot represents 1 biological replicate. The p values were calculated by two-way ANOVA with *Tukey's* multiple comparisons test (A).

Supplementary Tables

Table S1 GO enrichment analysis of the upregulated YY1-bound genes after YY1 depletion in $CD31^{+}$ ECs from ischemic limbs of *Cdh5-Cre;Yy1*$^{fl/+}$ mice compared to that of *Yy1*$^{fl/fl}$ controls 7 days after femoral artery ligation.

Pathway	P-value	Genes
GO:0045087~innate immune response	5.95E-16	ZBP1, CX3CR1, CSF1R, CD84, CLEC4A2, FCER1G, IFI204, CLEC4N, CD180, LY86, PLD4, TREM2, IFI202B, MCOLN2, LY9, LGALS3, HCK, PSTPIP1, CLEC4D, AIM2, CD14, SLC15A3
GO:0048246~macrophage chemotaxis	6.25E-07	CX3CR1, LGALS3, CCL12, GPR35, MMP9
GO:0007159~leukocyte cell-cell adhesion	5.40E-06	PTPRC, ITGAM, CCL5, ITGB2, FERMT3
GO:0001819~positive regulation of cytokine production	2.04E-05	CLEC4N, NFAM1, SLC11A1, HILPDA, CASP1, CD14
GO:0002250~adaptive immune response	5.21E-05	ADGRE1, CX3CR1, CD84, CLEC4D, TEC, CLEC4A2, CLEC4N, H2-DMA, GPR183, LILRB4A, MCOLN2

Table S2 GO enrichment analysis of the downregulated YY1-bound genes after YY1 depletion in $CD31^{+}$ ECs from ischemic limbs of *Cdh5-Cre;Yy1*$^{fl/+}$ mice compared to that of *Yy1*$^{fl/fl}$ controls 7 days after femoral artery ligation.

Pathway	P-value	Genes
GO:0006338~chromatin remodeling	0.022907	YY1, NUAK2, KSR1, NCOA3, PPM1H
GO:2000036~regulation of stem cell population maintenance	0.025544	NFYA, NCOA3
GO:0061028~establishment of endothelial barrier	0.033394	RAPGEF2, ICAM1
GO:0006357~regulation of transcription by RNA polymerase II	0.039665	YY1, CSRNP1, ZFP46, NFYA, ZFP426, PPM1H, SOX13
GO:0016477~cell migration	0.090994	FMNL1, SDC3, CDH23

Table S3 siRNA sequences used in this study

Human genes	siRNA sequence (5'-3')
YY1-1345	GAAGAUGAUGCUCCAAGAATT<br>UUCUUGGAGCAUCAUCUUCTT
YY1-1665	UCAGUCAACUAACCUGAAATT<br>UUUCAGGUUAGUUGACUGATT
SETD1A-2081	GUCCCUUCCUCUUGGUUAUTT<br>AUAACCAAGAGGAAGGGACTT
SETD1A-4477	GCAGUGAGUUUGAACAGAUTT<br>AUCUGUUCA AACUCACUGCTT
SETD1A-5209	CCCAGAAGAAGAUCGUGAUTT<br>AUCACGAUCUUCUUCUGGGTT
MAML1-1924	CAGCAGCUGUCCCAUAUAATT<br>UUAUAUGGGACAGCUGCUGTT
MAML1-1770	GCCACCGAGUAACUUGAAUTT<br>AUUCAAGUUACUCGGUGGCTT
MAML1-2435	CUGGGAAUCUGAUGCCAAUTT<br>AUUGGCAUCAGAUUCCCAGTT

Mouse genes	siRNA sequence (5'-3')
Axl-361	GAAUAUCACAGGUGCCAGATT<br>UCUGGCACCUGUGAUAUUCTT
Axl-1373	CUUAUGGAUAUAGGGCUAATT<br>UUAGCCCUAUAUCCAUAAGTT
Axl-1659	GGAAGAAGGAGACUCGAUATT<br>UAUCGAGUCUCCUUCUUCCTT
Epha2-1342	GAGCCCCACAUGAACUAUATT<br>UAUAGUUCAUGUGGGGCUCTT

Epha2-2230	GCGCUAGACAAGUUCCUUATT<br>UAAGGAACUUGUCUAGCGCTT
Epha2-1428	CGUCAGUAUUAACCAAACATT<br>UGUUUGGUUAAUACUGACGTT
Egfr-1323	CUCUCCAUAAAUGCUACAATT<br>UUGUAGCAUUUAUGGAGAGTT
Egfr-1444	CACGAGAACUAGAAAUUCUTT<br>AGAAUUUCUAGUUCUCGUGTT
Egfr-2293	GACGUCACAUUGUUCGAAATT<br>UUUCGAACAAUGUGACGUCTT

Table S4 Flow cytometry antibodies used in this study

Antibodies	Source	Cat No.	Dilution
APC anti-mouse CD3 Antibody	Biolegend	100235	1:100
FITC anti-mouse CD3 Antibody	Biolegend	100203	1:100
APC anti-mouse CD4 Antibody	Biolegend	116013	1:100
PE anti-human CD2 Antibody	Biolegend	300208	1:100
PE anti-mouse CD45 antibody	Biolegend	103106	1:100
APC anti-mouse CD31 Antibody	Biolegend	102510	1:100
Annexin V	Invitrogen	11-8005-72	1:50

Table S5 Immunostaining antibodies used in this study

Antibodies	Source	Cat No.	Dilution
Purified anti-mouse CD31	Biolegend	102502	1:100
Goat anti-Rat IgG (H+L) Alexa Fluor 488	Invitrogen	A-11006	1:1000

Table S6 Western blot antibodies used in this study

Antibodies	Source	Cat No.	Dilution
mouse anti-human/mouse β-actin	GenScript	A00702	1:2000
rabbit anti-human/mouse YY1	CST	46395S	1:2000
rabbit anti-human/mouse SETD1A	CST	50805	1:1000
rabbit anti-human/mouse SETD1B	CST	44922	1:1000
rabbit anti-human/mouse MLL1(N')	CST	14689	1:1000
rabbit anti-human/mouse MLL1(C')	CST	14197	1:1000
rabbit anti-human/mouse MLL2	CST	63735	1:1000
rabbit anti-human/mouse WDR5	CST	13105	1:1000
rabbit anti-human/mouse WDR82	CST	99715	1:1000
rabbit anti-human/mouse MENIN	CST	6891	1:1000
rabbit anti-human/mouse RNA Polymerase II	Active Motif	39097	1:1000
mouse anti-human/mouse p300	Active Motif	61903	1:1000
rabbit anti-mouse RBPSUH	CST	5313S	1:1000
rabbit anti-human/mouse MAML1	CST	12166S	1:1000
rabbit anti-mouse EZH2	CST	5246S	1:1000

Table S7 co-IP antibodies used in this study

Antibodies	Source	Cat No.	Dilution
mouse anti-human/mouse YY1	Active Motif	61779	1:100
mouse anti-mouse IgG antibody	Millipore	12-371	1:1000

Table S8 qPCR primers used in this study.

Genes	Forward	Reverse
Human		
YY1	GGAGGAATACCTGGCATTGACC	CCCTGAACATCTTTGTGCAGCC
MAML1	CCCCAGTGAGTCATTTCCTCT	GAGGTTGCTTTGCGATATGGA
SETD1A	TCGAGAGGAAGCTGTGGATACC	CGCCATCTGAGTCAGCATACAG
ACTB	CTTCCAGCCTTCCTTCCTGG	CTGTGTTGGCGTACAGGTCT
Mouse		
Yy1	CAGTGGTTGAAGAGCAGATCAT	AGGGAGTTTCTTGCCTGTCAT
Rbp/j	TGGCTACATCCATTACGGGCAG	GTGGAGTTGTGATACAGGGTCG
Maml1	CGTAGCTCAGAGCAACCTCAT	TTCATGTCTTCGTCGGGCAC
Hes1	GGAAATGACTGTGAAGCACCTCC	GAAGCGGGTCACCTCGTTCATG
Hey1	CAGTGCTACAGGATGACGGCTT	CCGTCCTAATGATGCGCTGCAG
Hey2	GCGAGACAACAGACCTCGTC	GGTGTCCCCTTGCACCTTAG
Cdkn1a	CCTGGTGATGTCCGACCTG	CCATGAGCGCATCGCAATC
Cdkn1b	TCAAACGTGAGAGTGTCTAACG	CCGGGCCGAAGAGATTTCTG
Ccnd1	GCAGAAGGAGATTGTGCCATCC	AGGAAGCGGTCCAGGTAGTTCA
Angpt1	AACCGAGCCTACTCACAGTACG	GCATCCTTCGTGCTGAAATCGG
Angpt2	AACTCGCTCCTTCAGAAGCAGC	TTCCGCACAGTCTCTGAAGGTG
Pdgfb	AATGCTGAGCGACCACTCCATC	TCGGGTCATGTTCAAGTCCAGC
Vegfa	CTGCTGTAACGATGAAGCCCTG	GCTGTAGGAAGCTCATCTCTCC
Hgf	GTCCTGAAGGCTCAGACTTGGT	CCAGCCGTAAATACTGCAAGTGG
Apln	AGGCATAGCGTCCTCACCTCTT	GGTGCAGAAACGACAAAGACGG
Ccl24	ATTCTGTGACCATCCCCTCAT	TGTATGTGCCTCTGAACCCAC
Gas6	TGCTGGCTTCCGAGTCTTC	CGGGGTCGTTCTCGAACAC

Grn	CTGCCCGTTCTCTAAGGGTG	ATCCCCACGAACCATCAACC
Areg	GGTCTTAGGCTCAGGCCATTA	CGCTTATGGTGGAAACCTCTC
Axl	GGTGTTTGAGCCAACCGTGGAA	GCCACCTTATGCCGATCTACCA
Epha2	GGCTGTACTCAAGTTTACCACCG	CCGCTTTCAGTGTCTTGATGGC
Egfr	GGACTGTGTCTCCTGCCAGAAT	GGCAGACATTCTGGATGGCACT
Actb	GGCTGTATTCCCCTCCATCG	CCAGTTGGTAACAATGCCATGT

Table S9 ChIP-seq and ChIP-qPCR antibodies used in this study

Antibodies	Source	Cat No.	Dilution
rabbit anti-human YY1	Active Motif	61779	1:50
rabbit anti-human H3K4me3	abcam	ab8580	1:100
rabbit anti-human H3K27ac	abcam	ab4729	1:100
rabbit anti-human/mouse MAML1	CST	12166S	1:100
rabbit anti-human/mouse SETD1A	CST	50805	1:100
mouse anti-human/mouse p300	Active Motif	61903	1:100
Normal Rabbit IgG	CST	2729	1:1000

Table S10 ChIP-qPCR primers used in this study.

Human genes	Forward	Reverse
MAML1	AGCGGGGCAGGAGGAAAA	GGCGAAGGCGCTCCATGA

Mouse genes	Forward	Reverse
Maml1	CCAGGCGCTCGGGAGACACA	ACCCGAAGTGGCAGCCGGCG
